# Ontogeny and phylogeny of the mammalian chondrocranium: the cupula nasi anterior and associated structures of the anterior head region

**DOI:** 10.1186/s40851-018-0112-0

**Published:** 2018-11-24

**Authors:** Evelyn Hüppi, Marcelo R. Sánchez-Villagra, Athanasia C. Tzika, Ingmar Werneburg

**Affiliations:** 1 Paläontologisches Institut und Museum der Universität Zürich, Karl-Schmid-Strasse 4, 8006 Zürich, Switzerland; 2 Laboratory of Artificial & Natural Evolution (LANE), Department of Genetics & Evolution, University of Geneva, Quai E. Ansermet 30, 1205 Genève, Switzerland; 3 Senckenberg Center for Human Evolution and Palaeoenvironment (HEP) at Eberhard Karls Universität, Sigwartstraße 10, 72076 Tübingen, Germany; 4 Fachbereich Geowissenschaften der Eberhard-Karls-Universität Tübingen, Hölderlinstraße 12, 72074 Tübingen, Germany; 5 Museum für Naturkunde, Leibniz-Institut für Evolutions- & Biodiversitätsforschung an der Humboldt-Universität zu Berlin, Invalidenstraße 43, 10115 Berlin, Germany

**Keywords:** Chondrocranium, Cupula nasi anterior, Mammalia, Ontogeny, Therian ancestor

## Abstract

**Background:**

The study of chondrocrania has a long tradition with a focus on single specimens and stages. It revealed great interspecific diversity and a notion of intraspecific variation. As an embryonic structure, the chondrocranium is subject to major changes in ontogeny with resorption and ossification of different cartilaginous structures. The cupula nasi anterior is the anteriormost portion of the cartilaginous nasal capsule and is expected to mirror much of the animal's life history and lifestyle. Its diversity in mammals is reflected in the external nasal anatomy of newborns. Marsupials and placentals show marked differences, likely related to breathing and suckling behavior.

**Results:**

We examined histological sections of five marsupial and three placentals species and traced the development of the cupula nasi anterior and the anterior nasal capsule. We found ontogenetic variation for nearly 50% of the 43 characters defined herein. By comparing to the literature and considering ontogenetic variation, we performed an analysis of character evolution in 70 mammalian species and reconstructed the nasal anatomy of the therian ancestor.

**Conclusions:**

At birth, marsupials have a complete but simple cupula nasi anterior, whereas placentals display a more diverse morphology due to reductions and variations of chondrocranial elements. The more compact nasal capsule in marsupials is related to a long and strong fixation to the mother’s teat after birth. Within marsupials and placentals, several derived characters distinguish major taxa, probably related to developmental and functional constraints. The reconstructed ancestral anatomy of the cupula nasi anterior supports the hypothesis that the therian ancestor was placental-like and that the marsupial lifestyle is more derived.

## Background

The chondrocranium is a transitory, embryological structure with biomechanical requirements in the developing head [1, 2]. It is enchondrally ossified [3] or resorbed in ontogeny [4]. Some of the cartilaginous elements continue to grow and differentiate after birth and are retained in adults, such as structures of the nasal region, including the cupula nasi anterior [2, 5]. Chondrocrania of several mammalian species were studied in the late 19th and in the twentieth century based on serial histological sections and whole-mount staining. These studies concerned mostly single specimens [6]; changes in ontogeny and individual variation [7] were rarely studied. Instead, the goal was, in most cases, to study the ‘stadium optimum’ of the cartilaginous skull [8], a subjectively defined stage, in which all chondrocranial structures are basically developed [9].

**Fig. 1:**
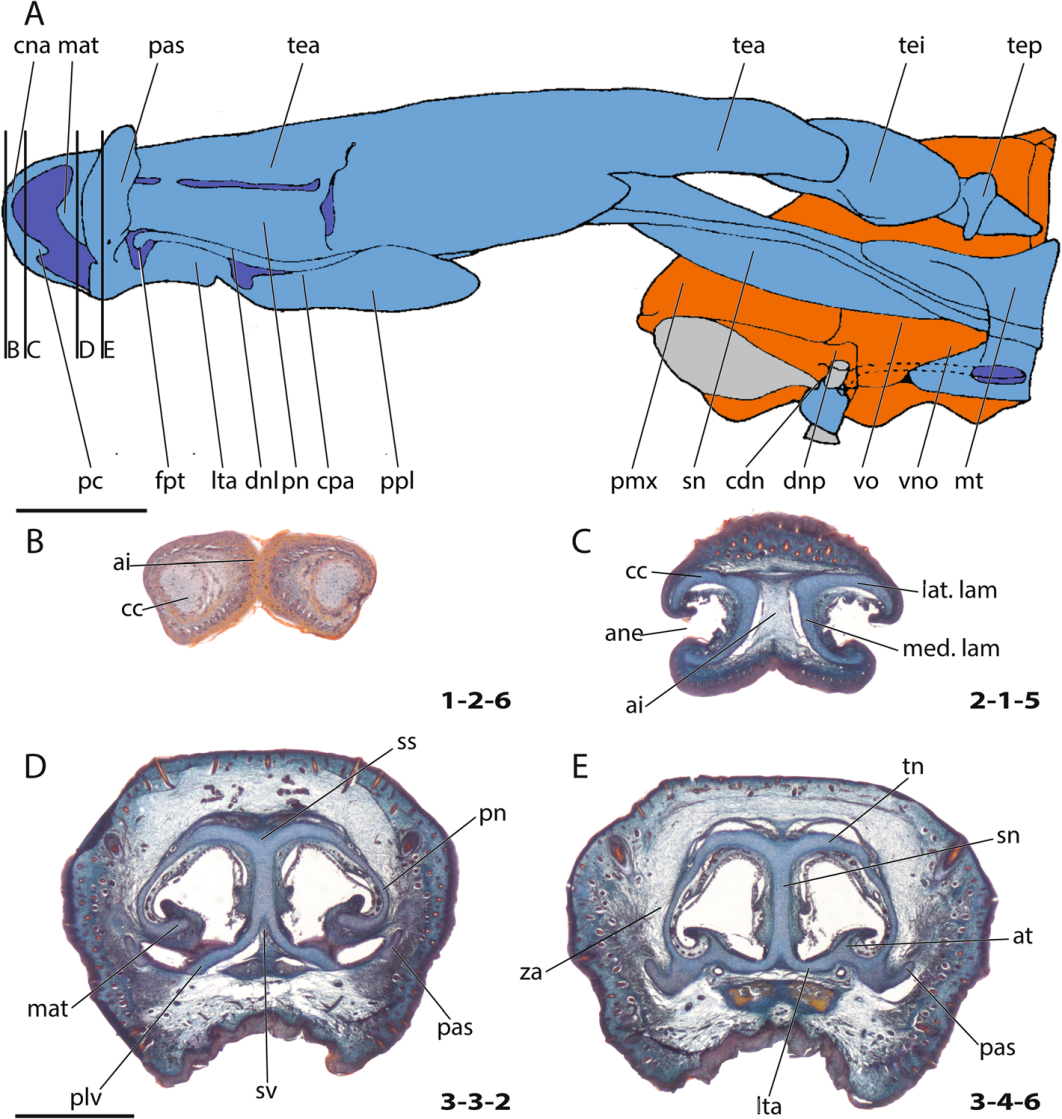
General anatomy of the cartilaginous nose. **a** Scheme adopted from Maier [56] on the rostral nasal cartilage in an adult *Neomys fodiens*. Cartilage in blue, bones (only on right body side) in orange, soft tissue in gray. Cross-sections of a pouch young *Caluromys philander*; dpn (postnatal days) 30, HL (head length) 13 mm at the ethmoidal region with details of the cupula nasi anterior, **b** anterior wall of cartilago cupularis, **c** cartilago cupularis in the region of fenestra narina, **d** region posterior to the fenestra narina, **e** anterior margin of the zona annularis. Numbers of histological serial sections are indicated at the bottom right of each figure. Numbers ascend in caudal direction. Scale bars equal 1 mm. Abbreviations: ai – area internarica, ane – apertura nasi externa, at – atrioturbinale, cc – cartilago cupularis, cdn – cartilago ductus nasopalatini, cna – cupula nasi anterior, cpa – cartilago paraseptalis anterior, dnl – ductus nasolacrimalis, dnp – ductus nasopaltinus, fpt – foramen praetransversale, lat. lam – lateral lamina, lta – lamina transversalis anterior, mat – marginoturbinale, med. lam – medial lamina, pas – processus alaris superior, pc – processus cupularis, plv – processus lateralis ventralis, pmx – praemaxillare, pn – paries nasi, ppl – processus paralacrimalis, sn – septum nasi, ss – sulcus supraseptalis, sv – sulcus ventralis, tea – tectum nasi anterius, tei – tectum nasi intermedium, tep – tectum nasi profundum, tn – tectum nasi, vno – organon vomeronasale, vo – vomer, za – zona annularis

The diversity of fully formed chondrocrania is stunning, particularly in the nasal region. Mammals are mostly macrosmatic, i.e. with a well-developed nasal region, and differ significantly in their nasal anatomy. The nasal capsule anatomy is expected to mirror much of an animal’s life history and ecology, as it is related to lactation of the young, olfactory communication, and different feeding adaptations of the snout, among other factors. The cupula nasi anterior is the rostral most portion of the chondrocranium and the nasal capsule (Fig. 1a) [4]. Some authors mentioned that the nasal skeleton is the part that ontogenetically changes least compared to the remainder of the chondrocranium [10]. Others have emphasized its great interspecific variability [11]. Although a well-developed cupula nasi anterior is common among Mammalia [12], in some species it is reduced [7, 13–15] or completely missing [16]. Whereas in placentals the cupula nasi anterior develops late relative to other cranial structures, or develops only when other parts of the chondrocranium are already resorbed [17, 18], a fully constructed cupula nasi anterior is a characteristic of marsupials at birth [19] and in early posthatching stages of monotremes [20], likely associated to their particular modes of lactation. Whereas monotremes slurp milk from milk fields on the mother’s belly, marsupials are tightly associated to the teats of the mother. Placentals suck from the mother’s teats but are not tightly fixed to them, which differentiates them from marsupial young in early postnatal life [21].

In adults, the cupula nasi anterior retains its size and form [5] as apertura piriformes [22] and surrounds the external nasal openings [23]. It supports the rhinarium [24], a glabrous and sensitive skin with mechanosensory functions [25] used for the tactile exploration of the environment [23].

**Fig. 2:**
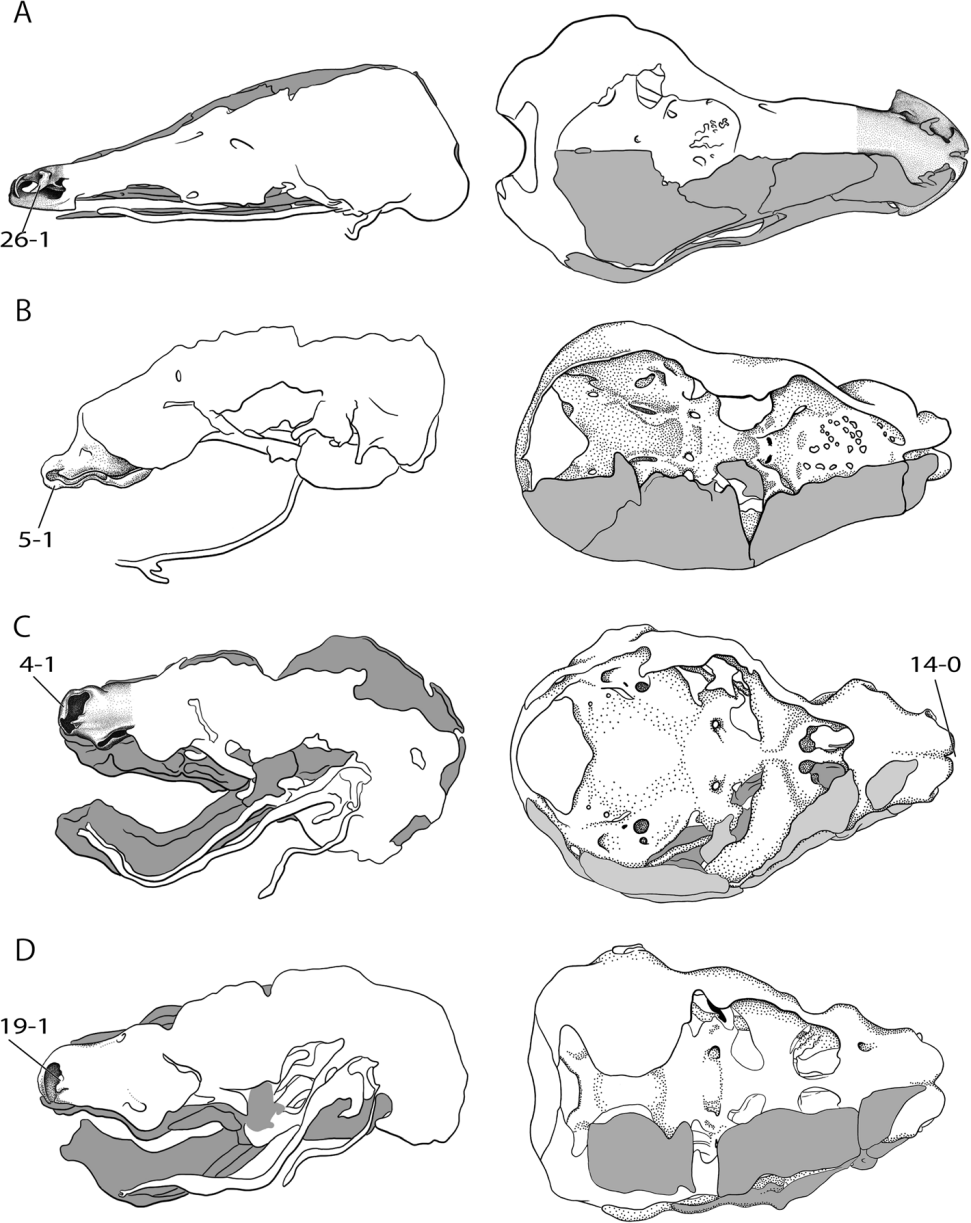
Overview of the variable shape of the cupula nasi anterior in mammals. Lateral view on the left, dorsal on the right. Cartilage colorless, bones in grey. Characters and character states are indicated (e.g., 26–1). **a** *Tachyglossus aculeatus*, HL 27 mm [20], **b** *Didelphis marsupialis*, CRL 45.5 mm [42], **c** *Wallabia rufogrisea*, HL 12 mm [54], **d** *Vombatus ursinus*, HL 14 mm [52]. Drawings by Timea Bodogán, modified from cited sources. Not to scale. Continued in Fig. 4

The rich and dispersed literature on the mammalian chondrocranium is not uniform in regard to methodology [19, 26, 27] and terminology; being largely descriptive, but some information on phylogenetic and ontogenetic differences in the cupula nasi anterior can be extracted. The true cupula nasi anterior includes the paired cartilago cupularis (Fig. 1b, c) with the lateral and medial lamina, the area internarica between them [24], the processus cupularis, and the processus lateralis ventralis [22, 28]. The lateral and medial lamina of the cartilago cupularis (Fig. 1c) are continuous with the tectum and the septum nasi of the nasal capsule [20]. The extent of the area internarica depends on the development of the anterior wall of the cartilago cupularis [24, 26] (Fig. 2c), and the depth of the area depends on the position of the anterior end of the septum nasi. The processus cupularis (Fig. 1a) connects ventrocaudally to the cartilago cupularis. Together with the processus lateralis ventralis (Fig. 1d), the projection that connects the cartilago cupularis with the lower anterior edge of the septum nasi, they surround the fenestra narina, the rostral opening of the nasal capsule [4, 28]. The floor below the cupula nasi anterior is formed by the cartilago cupularis, the processus cupularis, and the processus lateralis ventralis.

**Fig. 3:**
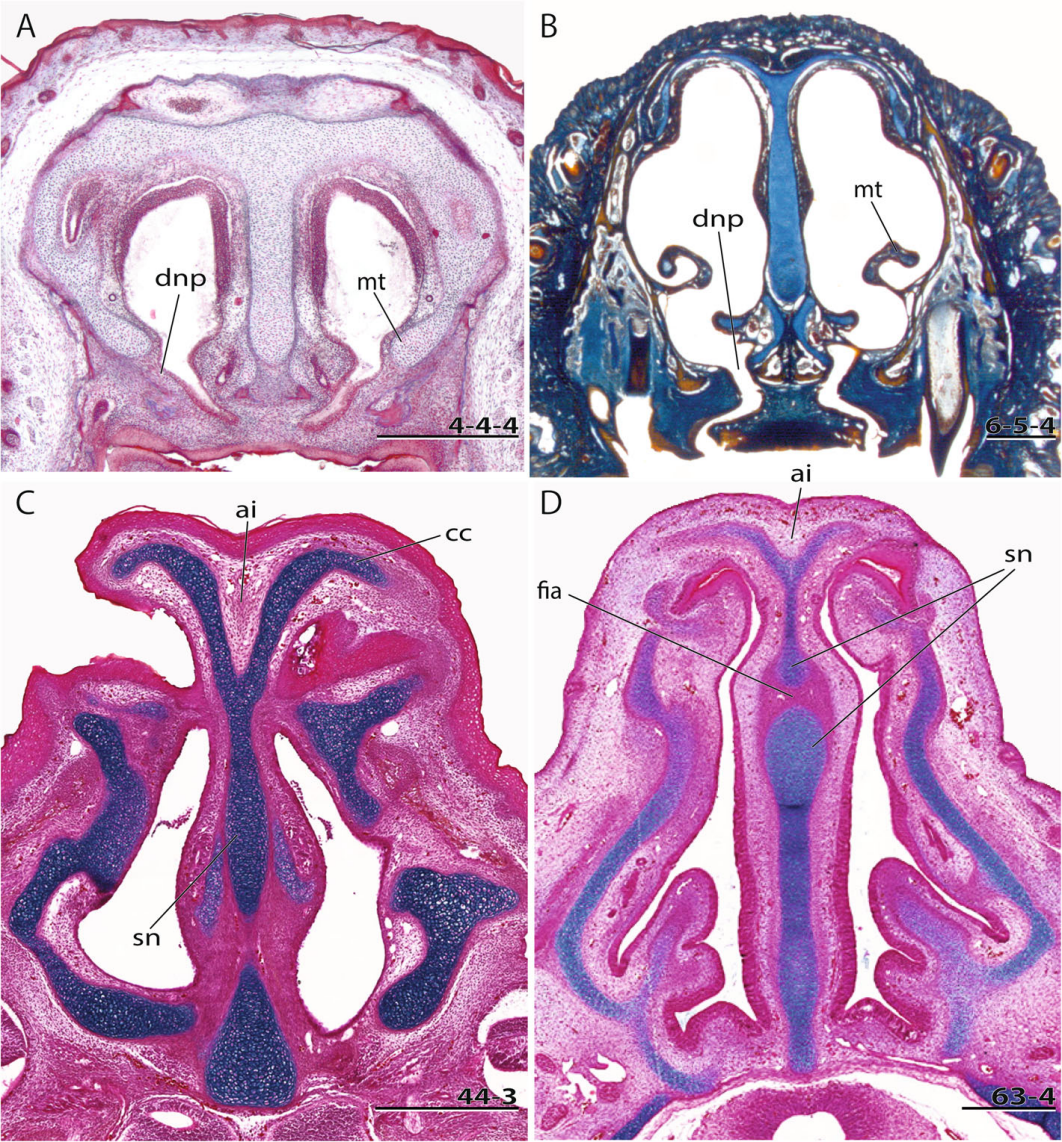
Cross sections of pouch young *Monodelphis domestica* where the ductus nasopalatinus opens to the nasal cavity, **a** CRL (crown-rump length) 11.5 mm, **b** CRL 63 mm. Transversal sections of the nasal capsule in (**c**) *Petaurus breviceps*, CRL 9 mm, and (**d**) *Atelerix albiventris*, dpc 25 (days post conception). Numbers of histological serial sections are indicated at the bottom right of each figure. Scale bars equal 500 μm. Continued in Fig. 5. Abbreviations: ai – area internarica, at – atrioturbinale, cc – cartilago cupularis, dnp – ductus nasopalatinus, fia – fenestra internasalis anterior, lat. Lam – lateral lamina, lta – lamina transversalis anterior, med. Lam – medial lamina, mt – maxilloturbinale, sn – septum nasi

**Fig. 4:**
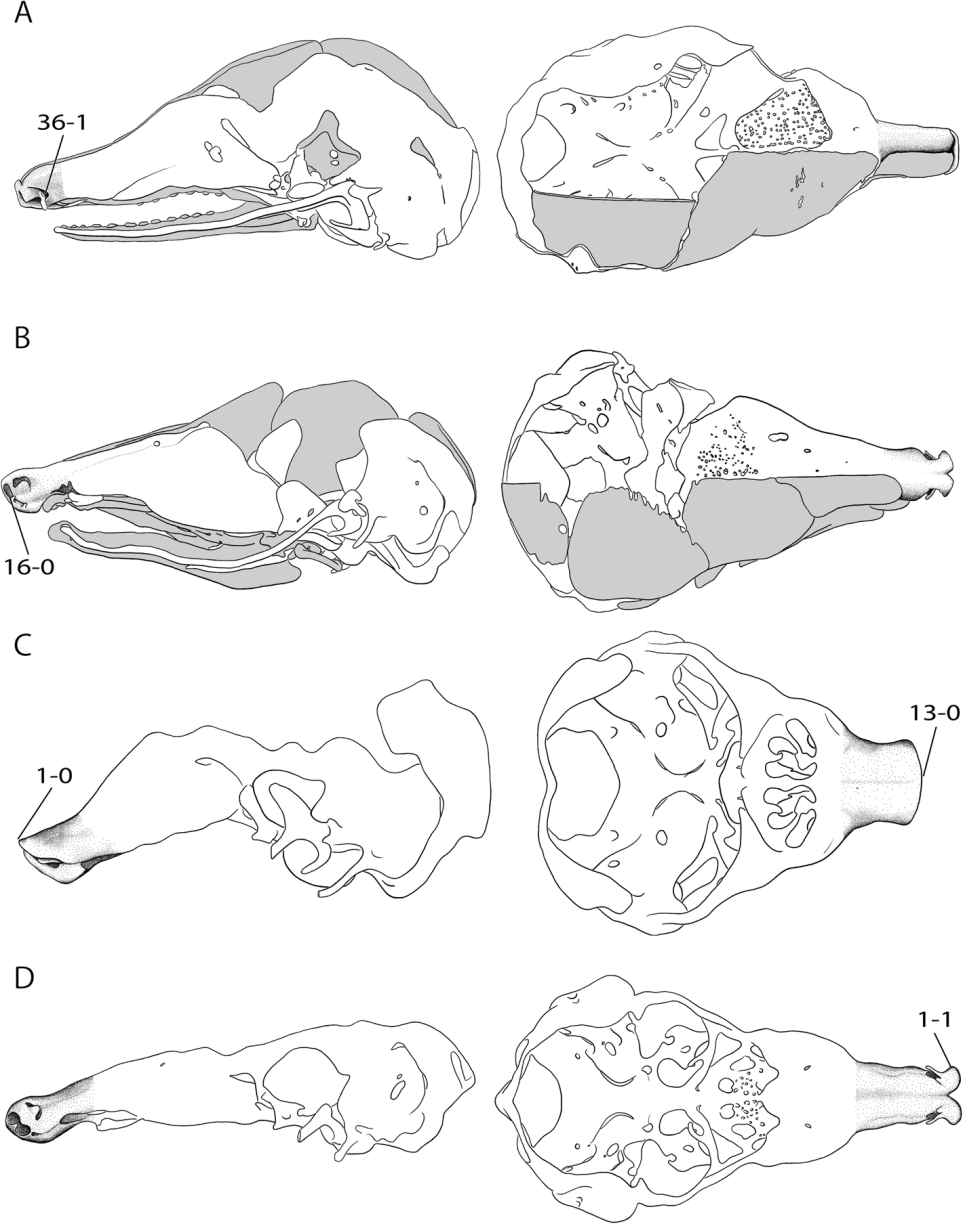
Overview of the variable shape of the cupula nasi anterior in mammals. Continued from Fig. 2. **a** *Dasypus novemcinctus*, CRL 40 mm [32], **b** *Hemicentetes semispinosus*, HL 14 mm [39], **c** *Eremitalpa granti*, CRL 28.5 mm [30], **d** *Setifer setosus*, CRL 20.4 mm [30]. Drawings by Timea Bodogán, modified from cited sources. Not to scale. Continued in Fig. 6

**Fig. 5:**
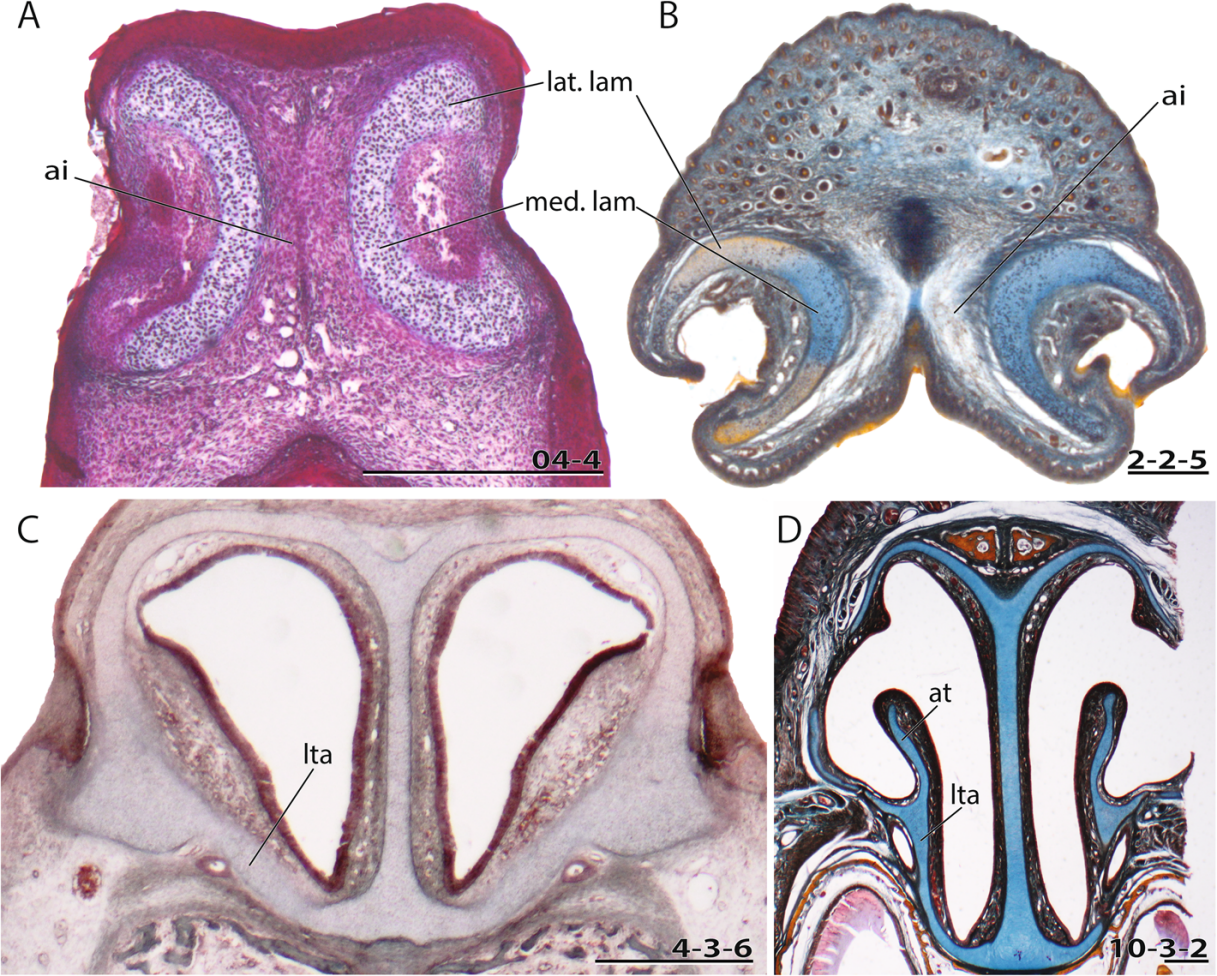
Continued from Fig. 3. Cross sections of a pouch young *Caluromys philander* at the region of the opening of fenestra narina, **a** HL 6 mm, **b** HL 26 mm. Cross sections of a pouch young *Macropus eugenii* at the anterior region of the lamina transversalis anterior, **c** HL 11 mm, **d** HL 53 mm. Scale bars equal 500 μm

In the anterior region of the nasal capsule, the septum nasi and the tectum nasi can be fenestrated, forming the fenestra internasalis (fenestration of septum nasi, Fig. 3d) and the fenestra superior nasi (fenestration of tectum nasi, Fig. 4a). The lamina transversalis anterior (Fig. 1e) is part of the solum nasi [22], separating fenestra narina and fenestra basalis. If fused with the septum nasi and the paries nasi, they together form the zona annularis (Fig. 1e), a ring-shaped cartilaginous structure enclosing the cavum nasi [29]. The processus alaris superior (Fig. 1e) originates from the ventrolateral edge of the anterior border of the lamina transversalis anterior and the paries nasi [20]. The position of the process in marsupials is conserved. In contrast, in placentals the position is variable [20]. De Beer [26] noted that the processus alaris superior is remarkably constant in tetrapods. Kuhn [20] suggested that, given the high variation in shape, position, and orientation of the external nares, it is understandably difficult to homologize the processes, which often have different names in the literature. The processus alaris superior supports the apertura nasi externa [10, 26] together with the processus cupularis [18], and its link to the atrioturbinale (Fig. 5d) serves to regulate the air passage at the entrance of the nasal cavity [24]. Processus alaris superior serves as attachment site for distinct facial musculature [4, 30]. In a few placentals [31], the processus is fused with processus cupularis, building the commissura alicupularis [29].

Concerning the turbinals, the inward-rolled, dorsal margin of the fenestra narina forms the marginoturbinale [24] (Fig. 1d). The atrioturbinale, which continues rostrally with the marginoturbinale and caudally with the maxilloturbinale, is formed by a fold of the lamina transversalis anterior [32, 33]. The maxilloturbinale (Fig. 3a, b) is formed by the inward-rolled ventral margin of the paries nasi posterior to the lamina transversalis anterior. Whereas the marginoturbinale and the atrioturbinale remain cartilaginous in adults, only the maxilloturbinale ossifies [34, 35].

The aims of our study were (i) to study ontogenetic variation of the cupula nasi anterior in selected mammalian species, (ii) to synthesize the available literature on the anatomy of the cupula nasi anterior among mammals, and (iii) to explore the potential phylogenetic signal of this region of the skull among mammals. First, we defined morphological characters and mapped them on the mammalian phylogeny. This allowed us to test the hypothesis whether specific characteristics are associated to particular phylogenetic and ecological groups. We focused in particular on the differentiation between marsupials and placentals and the ancestral therian pattern.

## Results

In total, 43 discrete characters with mainly binary character states were defined to characterize the anatomy of the cupula nasi anterior, the lamina transversalis anterior, and the turbinals of the anterior nasal capsule. One monotreme, 14 marsupial, and 55 placental species were coded, including literature resources. Ontogenetic variation was documented for four marsupial species. The literature is limited on the ontogenetic variation in placental species.

A character matrix with all specimens, including different ontogenetic stages of marsupial species, is provided in Appendices 1–2. Based on our own observations and on literature information on other mammals, we defined three categories of characters. The first category represents clear states such as absence or presence of a structure. It includes characters 1, 4, 8, 10, 11, 13, 15, 16, 19, 20, 27, 29, 32, 33, 34, 35, 36, 37, 39, 40, 41, and 43. The second category represents characters of which state definition is relative such as a large or a small appearance and includes characters 3, 9, 12, and 21. The third category represents characters with detected ontogenetic variation in marsupials and includes characters 2, 5, 6, 7, 14, 17, 18, 22, 23, 24, 25, 26, 28, 30, 31, 38, and 42. These categories are considered when interpreting character evolution.

### [1] Cupula nasi anterior

#### Definition

The cupula nasi anterior can be absent (0) or present (1).

#### Discussion

In all marsupials, cupula nasi is completely developed at the time of birth. A pre-cartilaginous cupula nasi is only reported for a dpc (days post conception) 14 *Monodelphis domestica* [36], and a missing cupula nasi for an intrauterine *Trichosurus vulpecula* [37]. In all stages of *Sminthopsis virginiae*, the cartilago cupularis is more extensive than in any other examined marsupial. It closes the nasal capsule not only to the front, but also to the side and forms a cavity between the fenestra narina and the tip of the nasal capsule.

Except for the dorsal portion, the cupula nasi anterior is chondrified in *Atelerix albiventris* at dpc 25, about 10 days before birth. A well-developed cupula nasi before birth has been reported for other eulipotyphlans. In contrast, in both stages of *Echinops telfairi* studied herein, the cupula nasi anterior is missing, like in a relative early stage of another afrosoricid, *Eremitalpa granti* [30] [CRL (crown-rump length) 28.5 mm] (Fig. 4c). By dpc 21, cupula nasi anterior is recognizable in *Acomys dimidiatus* at its pre-cartilaginous stage, whereas the tectum nasi, the paries nasi, and the septum nasi are well chondrified. The condition that the cartilago cupularis and its anterior closure chondrify relatively late is also reported for other placentals, e.g., *Peromyscus maniculatus* [28], *Castor fiber* [38], and *Eremitalpa granti* [30]. In *Loxodonta africana* [25], as in Catarrhini, many structures of the cupula nasi anterior are reduced.

#### Evolution

The cupula nasi anterior is present in most mammalian species. The only species reported to lack cupula nasi is the catarrhine primate *Papio hamadryas* [16], but this is most likely due to sampling of a stage too early to possess this structure.

### [2] Cupula nasi anterior: Robustness

#### Definition

The cartilage of the cupula nasi anterior can be delicate (0) or robust (1).

#### Discussion

**Fig. 6:**
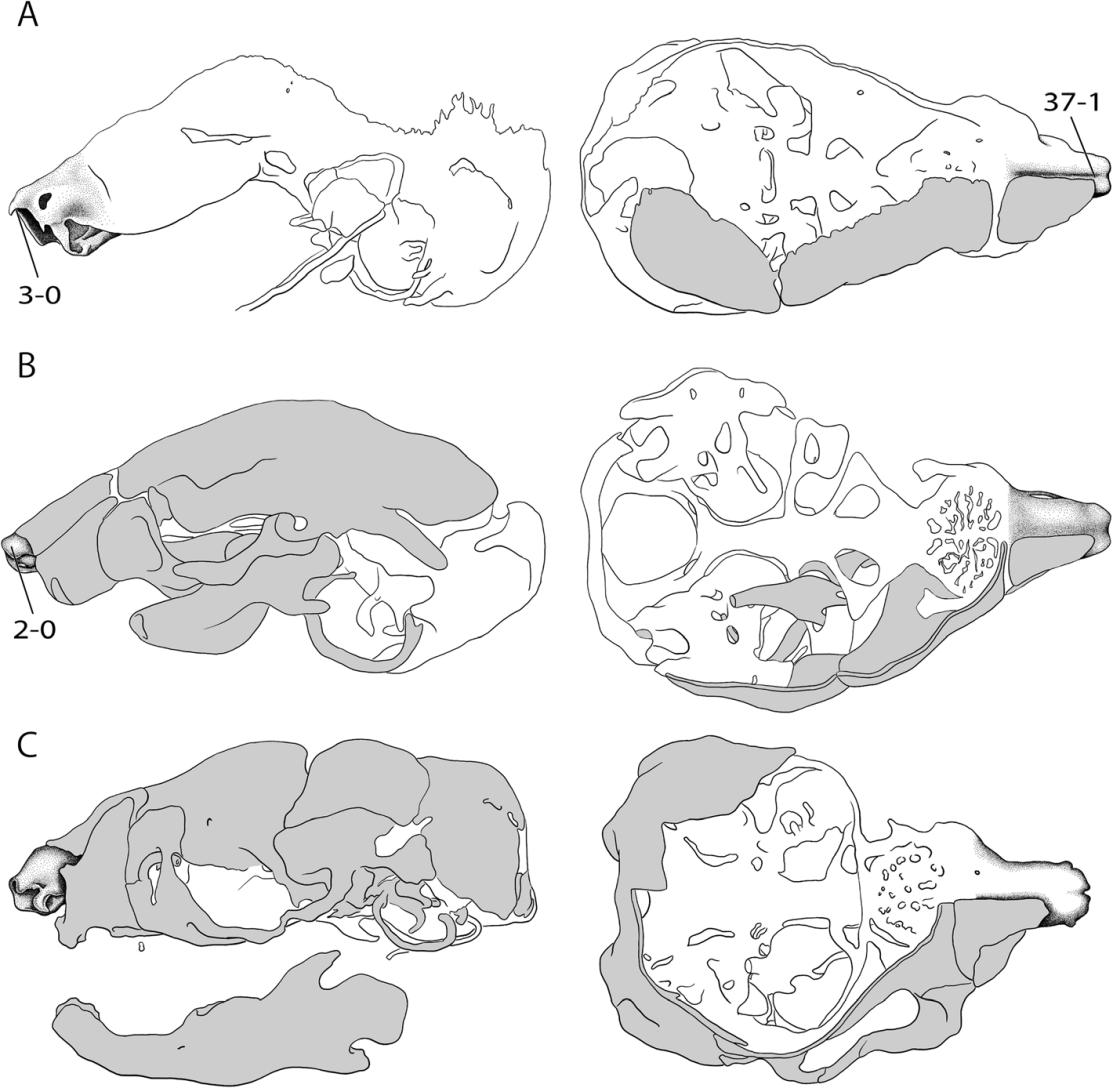
Overview of the variable shape of the cupula nasi anterior in mammals. Continued from Fig. 4. **a** *Orycteropus cuniculus*, CRL 45 mm [8], **b** *Octodontomys gliroides*, HL 19 mm [22], **c** *Phodopus sungorus*, HL 11.5 mm [46]. Drawings by Timea Bodogán, modified from cited sources. Not to scale. Continued in Fig. 7

In all ontogenetic series of marsupials examined, the robust cartilage of the earliest stages becomes more delicate during ontogeny. In placentals, the same developmental progress with a more delicate cartilage (Fig. 6b) in later stages is reported for rodents, such as *Acomys dimidiatus* [28].

#### Evolution

The ancestral condition of cartilage robustness is not certain. Afrotheria as a whole is characterized by a robust cupula nasi anterior with the exception of *Hemicentetes semispinosus* [39]. The same is true for Sciuromorpha, whereas a delicate condition is found in Macropodidae, Muroidea, and Octodontidae.

### [3] Cupula nasi anterior: Size

#### Definition

The cartilago cupularis can be small (i.e., reduced in some parts) (0) or large (i.e., complete cartilago cupularis) (1).

#### Discussion

A reduction of the cartilago cupularis is only known for placentals (Fig. 6a).

#### Evolution

The ancestral condition is uncertain. All marsupials, however, have a large and well-developed cartilago cupularis. Among placental mammals, the same condition is found in Erinaceidae. Ancestrally, Euarchontoglires have a small cartilago cupularis, although within Rodentia, particularly in Myomorpha, the size varies.

### [4] Rostral cartilago cupularis

#### Definition

Rostrally, cartilago cupularis can be open (0) or closed (1) (= character 1 of Freyer [36]).

#### Discussion

A cupula nasi anterior with a well-developed anterior wall is present in all marsupials (Fig. 2b–d). In *Perameles* sp. [40], a reduced anterior closure is defined by the lateral-oral opening of the fenestra narina. In *Macropus eugenii,* the anterior wall is relatively smaller in the later stages, as it is also described for *Isoodon obesulus* [41]. In all other marsupials, the anterior wall persists throughout ontogeny.

**Fig. 7:**
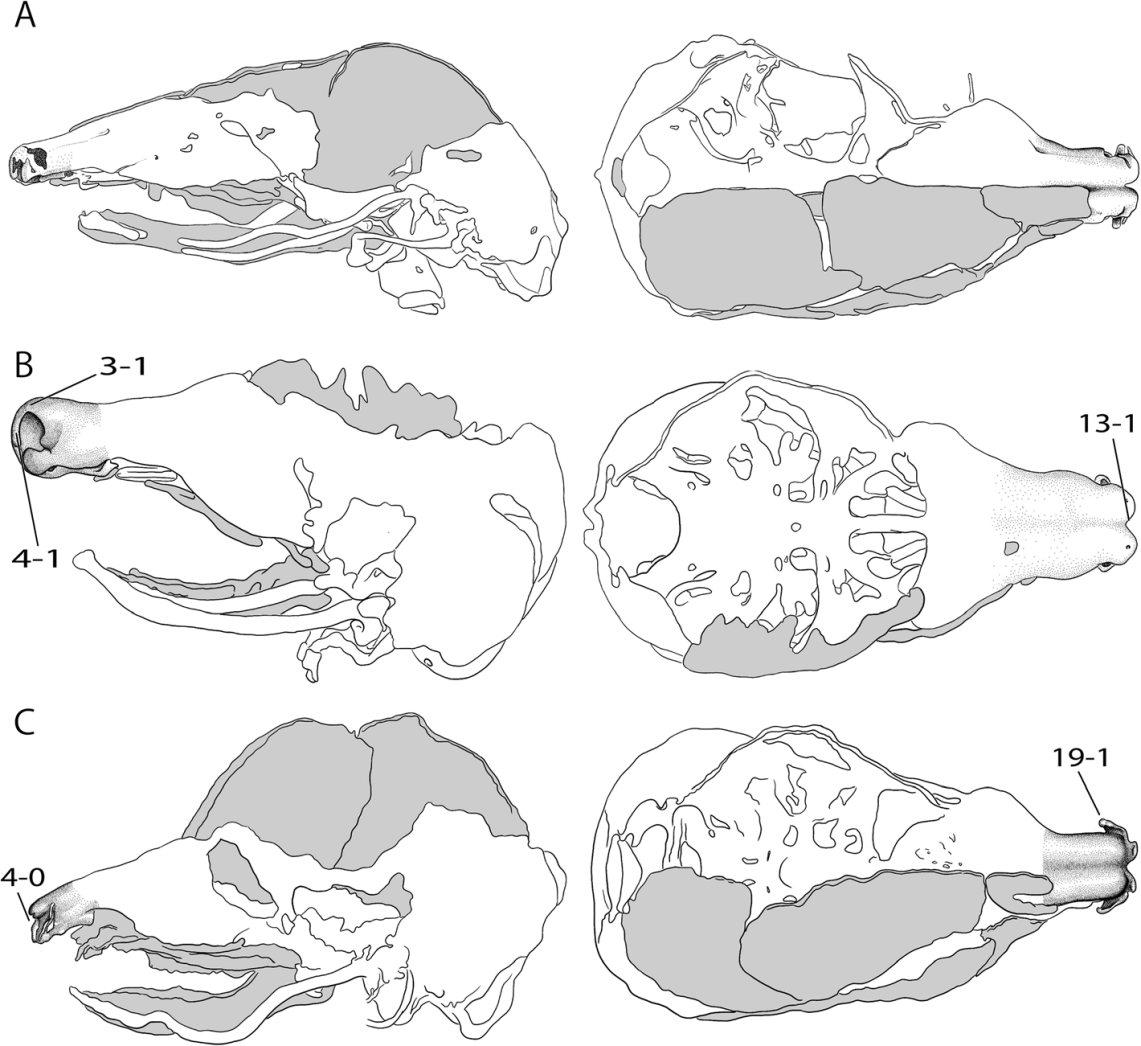
Overview of the variable shape of the cupula nasi anterior in mammals. Continued from Fig. 6. **a** *Ptilocercus lowii*, CRL 45 mm [89], **b** *Erinaceus europaeus*, CRL 19 mm [6], **c** *Cryptoprocta ferox,* CRL 54 mm [15]. Drawings by Timea Bodogán, modified from cited sources. Not to scale

In placentals, a cupula nasi anterior without anterior wall (Fig. 7c) is common. It occurs as cartilago cupularis that closes the cupula nasi anterior rostrally (Fig. 7b). In some placentals, such as *Octodon degus* [22], the anterior wall develops in later stages, whereas in other species, e.g., *Sciurus vulgaris* [38], the anterior wall is already developed at the earliest stage.

#### Evolution

Ancestrally, cartilago cupularis was closed in Mammalia. It opens in Atlantogenata with only *Procavia capensis* [25] and *Setifer setosus* [30] showing the closed condition. One marsupial, *Perameles* sp. [40], and several placentals, namely Lagomorpha, *Minopterus schreibersi* [31], Catarrhini, *Bos taurus* [6], Carnivora, *Jaculus jaculus* [28], Octodontidae, and Cricetida, have an open condition. Only one reversal to a closed condition is recorded, for *Aconaemys fuscus* [22] within Octodontidae.

### [5] Processus cupularis

#### Definition

The processus cupularis can be absent (0), present (1), or masked (2) (= character 25 of Frahnert [38] and character 3 of Freyer [36]). Synonyms: Processus alaris inferior of Gaupp [29], processus alaris medianus of Fawcett [6], processus anterior of Fischer [10].

#### Discussion

In marsupials, a small processus cupularis is present in most specimens in late ontogeny. In *Didelphis marsupialis* [42], a process originates from the ventral portion of the cartilago cupularis and projects in the direction of the processus alaris superior (Fig. 2b). For that reason, it is indeed the same structure despite the different name “processus alaris inferior”.

**Fig. 8:**
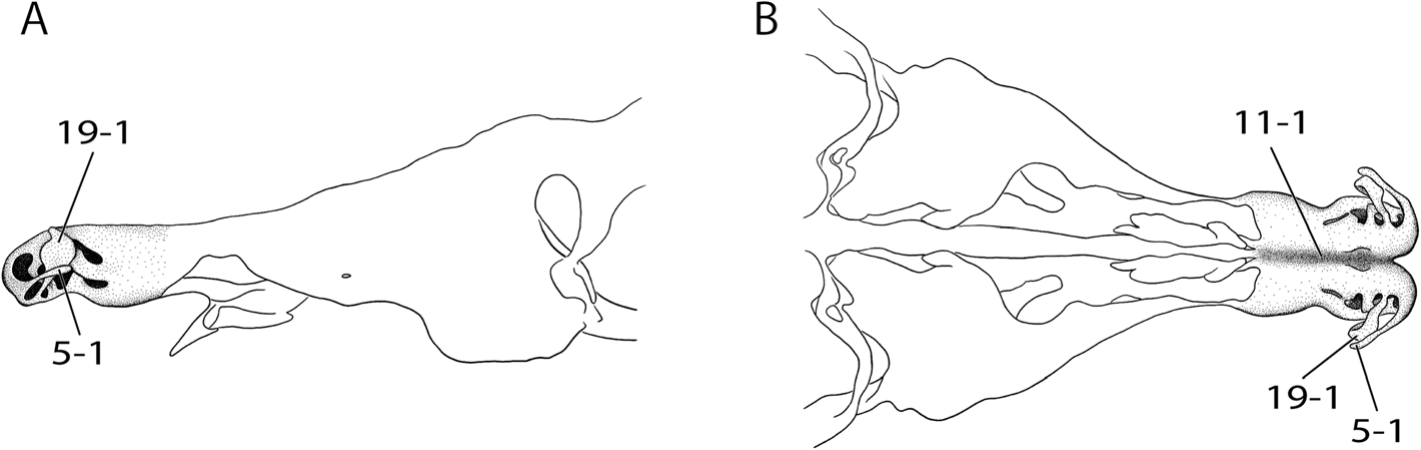
Overview of the processes of the nasal capsule in *Suncus orangiae*, CRL 15.6 mm [30]. Lateral view on the left, ventral on the right. **a** Processus cupularis, processus alaris superior, and (**b**) sulcus ventralis. Drawings by Timea Bodogán, modified from cited source. Not to scale

Most placentals have a processus cupularis (Fig. 8a). In *Atelerix albiventris*, it is masked due to a similar breadth of the ventral part of cartilago cupularis and processus lateralis ventralis. The only reported exception is *Eremitalpa granti* [30] (CRL 45 mm), where it originates from the processus lateralis ventralis.

#### Evolution

The ancestral mammalian condition is the absence of the processus cupularis. Afroinsectivora have this process. A masked process occurs independently in different species.

### [6] Medial lamina

#### Definition

The medial lamina can be shorter (0) or longer (1) than the lateral lamina, or have the same length (2).

#### Discussion

In early marsupial stages, the relatively long medial lamina of the cartilago cupularis gives the cupula nasi anterior a rectangular appearance in frontal view that reflects the compact snout of the early pouch young, which has about the same height as the remainder of the chondrocranium. The proportions of both laminae change through ontogeny (Fig. 5a, b), the lateral laminae becoming relatively long in relation to the medial lamina. The longer lateral laminae give the cartilago cupularis a rounder shape in cross section. The shape and the extent of the changes vary among the examined species. *Macropus eugenii* is the only examined species in which these changes were not observed, as the angular shape persists in late stages. In most placentals, the medial lamina is shorter than the lateral one.

#### Evolution

Ancestrally, the medial lamina is shorter than the lateral lamina in Mammalia. Among marsupials, Phalangerida show a longer medial lamina. The same is true for a few other marsupial and a few placental species. Both laminae have the same length only in *Castor fiber* [38] and in early stages of *Macropus eugenii*.

### [7] Dorsal lateral lamina

#### Definition

The dorsal lateral lamina can be longer (0) or shorter (1) than the ventral lamina, or have the same length (2).

#### Discussion

In marsupials, only in the early stages of *Macropus eugenii* and *Sminthopsis virginiae* are the lateral laminae longer than the ventral ones. Changes in ontogeny are common, except for *Monodelphis domestica*. The length of the lateral laminae among placentals is variable.

#### Evolution

Ancestrally in mammals, the dorsal lateral lamina is longer or has the same length as the ventral lamina. In Placentalia, it is longer. Within the latter, both laminae have the same length in Scrotifera, with only *Minopterus schreibersi* [31] having a shorter lateral lamina. Some terminal taxa show changes from the placental ground pattern or, within marsupials, show some variation in ontogeny.

### [8] Processus lateralis ventralis

#### Definition

The processus lateralis ventralis can be absent (0) or present (1) (= character 26 of Frahnert [38]). Synonym: Processus laterales anteriores of Fawcett [6].

#### Discussion

A processus lateralis ventralis is present in all examined marsupials to a different extent, but absent in some placentals and in monotremes [11].

#### Evolution

Ancestrally, the processus lateralis ventralis is present in Mammalia. It is absent only in *Oryctolagus cuniculus* and *Loxodonta africana*.

### [9] Processus lateralis ventralis: Breadth

#### Definition

The breadth of the processus lateralis ventralis can be less than half (narrow) (0) or half of the breadth of the cartilago cupularis (broad) (1).

#### Discussion

In *Macropus eugenii* and *Caluromys philander*, the process becomes broader in ontogeny, whereas in the other marsupial ontogenetic series the relation of the process to the cartilago cupularis remains the same. The processus lateralis ventralis is broad in most marsupials, except in Diprotodontia.

In placentals, few species have a narrow process, as exemplified by a pre-cartilaginous stage in *Acomys dimidiatus*. *Atelerix albiventris* has a processus lateralis ventralis that is nearly as broad as cartilago cupularis.

#### Evolution

Ancestrally, processus lateralis ventralis is half of the breadth of cartilago cupularis. Only a few species, including *Homo sapiens* [43, 44], have a narrow process.

### [10] Processus lateralis ventralis: Connection

#### Definition

Processus lateralis ventralis is either not continuously connected with the lamina transversalis anterior (0), or it is continuously connected with the lamina transversalis anterior (1) (= character 4 of Freyer [36]).

#### Discussion

In most marsupials, the lamina transversalis anterior is at the same level as processus lateralis ventralis and forms a continuous rostral floor. The two exceptions are one stage of *Monodelphis domestica* (this study) and *Perameles nasuta* [41], as their process lies inferiorly to the lamina transversalis anterior.

Most placentals have a continuous connection between the process and the lamina, including *Atelerix albiventris* and *Echinops telfairi*. In *Erinaceus europaeus*, stages with ([45]: figure 2) and without ([6]: plate II) continuous connection have been described.

#### Evolution

The mammalian ancestor is reconstructed to have the processus lateralis ventralis continuously connected with the lamina transversalis anterior. It is loosely connected in Primates and Hystricomorpha and not continuously connected in a few species, including *Orycteropus afer* [25], *Miniopterus schreibersi* [31], *Perameles nasuta* [41], and *Acomys* sp. [28].

### [11] Sulcus ventralis

#### Definition

The sulcus ventralis can be absent (0) or present (1). Synonym: Sulcus subseptalis of Hauck [46].

#### Discussion

*Caluromys philander* lacks the sulcus in the early stages studied. In the other marsupials so far studied, the presence or absence of sulcus ventralis is stable through ontogeny. Literature information on ontogenetic changes in placentals is not available.

#### Evolution

The sulcus ventralis (Fig. 8b) is present in all mammals except for Didelphidae, *Petaurus breviceps*, and *Jaculus jaculus* [28].

### [12] Floor of cupula nasi anterior

#### Definition

The floor of cupula nasi anterior is half covered (0), completely covered (1), or incompletely covered (less than half) (2) by the cartilago cupularis and the processus lateralis ventralis.

#### Discussion

**Fig. 9:**
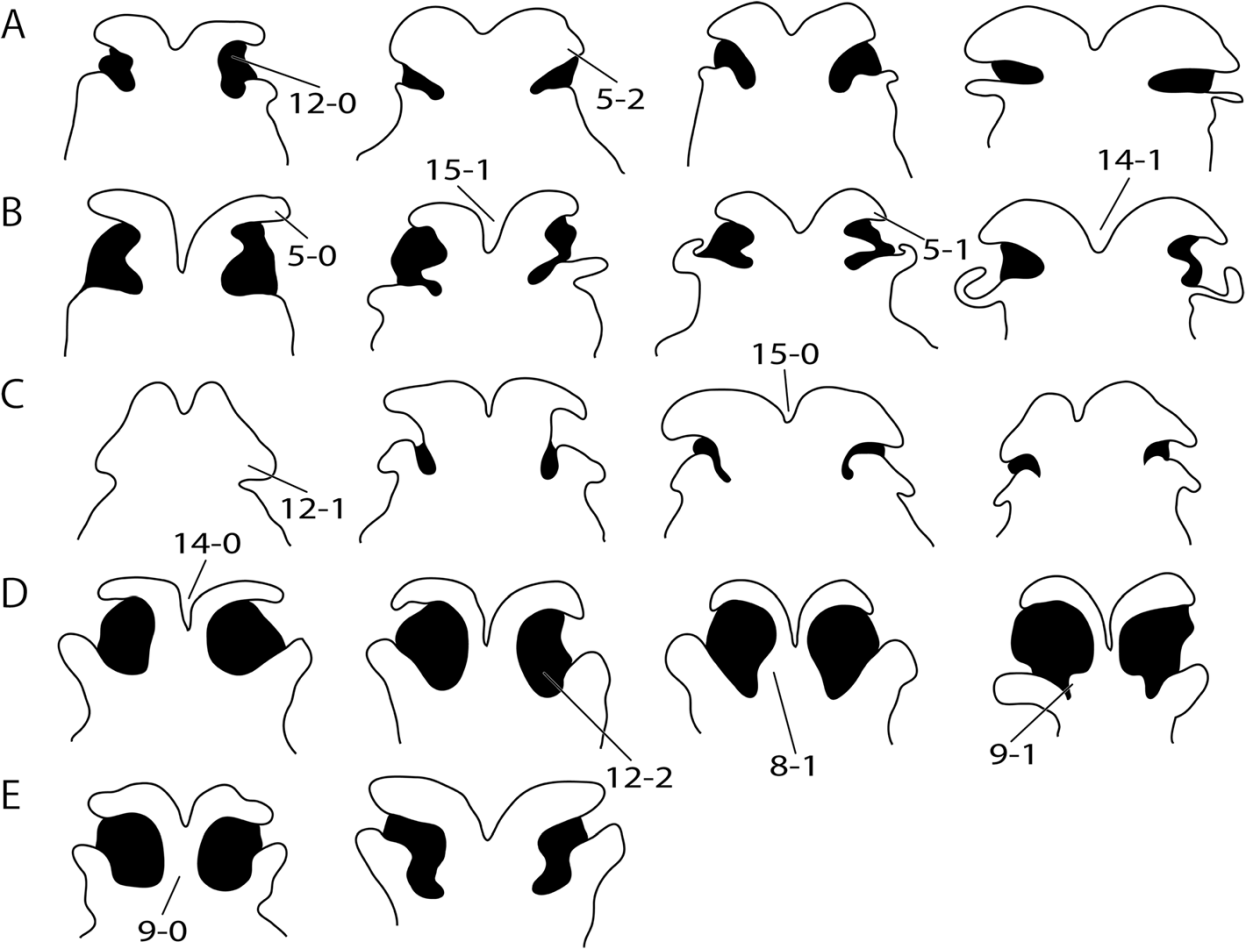
Ventral view of the cupula nasi anterior in ontogenetic series of marsupials. Characters and character states are indicated. Ontogenetic stages increase from left to right. **a** *Monodelphis domestica*: CRL 10.5 mm (ESUT-Mo10.5), 11.5 mm (ESUT-Mo11.5), 20.5 mm (ESUT-Mo8.5) and 63 mm (ESUT-Mo63), **b** *Caluromys philander*: HL 6 mm (ESUT-C6), 7.5 mm (ESUT-C15), 13 mm (ESUT-C13) and 26 mm (ESUT-C26), **c** *Sminthopsis virginiae*: HL 3.5 mm (AMNH SR 1A), 4.5 mm (AMNH SR 2A), 9 mm (AMNH SR 3A) and 11.5 mm (AMNH SR 4A), **d** *Macropus eugenii*: HL 11 mm (ESUT-M11), 16 mm (ESUT-M16), 28 mm (ESUT-M28) and 53 mm (ESUT-M53), **e** *Petaurus breviceps*: CRL 9 mm (LANE-P82A) and 11 mm (LANE-P48). Drawings from 3d-reconstructions. Not to scale

Most marsupials have a rather complete ventral portion of the cupula nasi anterior (Fig. 9a–c), except for diprotodontians (Fig. 9d, e). Reasons for the incomplete floor are the missing ventrolateral laminae of cartilago cupularis and a narrow processus lateralis ventralis. In some species, the completeness of the floor appears to be related to the position of the premaxilla, situated ventrally to the nasal capsule. In *Macropus eugenii*, the anterior margin of the premaxilla reaches up to the middle of the fenestra narina, as in other diprotodontians. In *M. domestica*, *C. philander*, and *Sminthopsis virginiae*, the anterior margin of the premaxilla is posterior to the fenestra narina, like in *Dasyurus viverrinus* [37] and *Perameles* sp. [40]. In *Isoodon obesulus* [41], the position of the premaxilla is between the two states described*.*

A complete floor inferior to the cupula nasi anterior has been reported for *Tachyglossus aculeatus* [20] and many placentals, such as *Atelerix albiventris*. In *Echinops telfairi* and *Acomys dimidiatus*, the ventral portion of the cartilago cupularis is not developed due to the late chondrification of the cupula nasi anterior.

#### Evolution

Ancestrally, Mammalia have a complete floor of cupula nasi anterior. Less than half of the cupula nasi anterior is covered by cartilage in Euarchontoglires, Diprontodontia, *Dasypus novemcinctus* [32, 33], and *Miniopterus schreibersi* [31]. Carnivora are apomorphically characterized by a half-covered floor. Large variation, in contrast, occurs within Rodentia.

### [13] Area internarica

#### Definition

Area internarica can be absent (0) or present (1). Synonyms: Spatium internasale of Kuhn [20], cavum internasale of Schunke and Zeller [39].

#### Discussion

The few species missing the cartilago cupularis do not have an area internarica (Fig. 4c).

#### Evolution

Area internarica is present in all mammals except for *Dasypus novemcinctus* [32, 33], *Perameles* sp. [40], and *Eremitalpa granti* [30].

### [14] Area internarica: Width

#### Definition

The area internarica can be narrow (0), when the width between the most anterior points of the cartilago cupularis to depth ratio is less than two, or wide (1) when the ratio is two or more.

#### Discussion

All stages of *Macropus eugenii, Petaurus breviceps* and *Sminthopsis virginiae* have a narrow area internarica. In *M. eugenii*, the width-to-depth ratio is smaller in the latest stage (0.99) compared to the other stages (from 1.15 to 1.94) (Table 1), whereas in *P. breviceps* (from 1.51 and 1.63) and *S. virginiae* (from 1.17 to 1.75), the ratio increases, but the area internarica remains narrow following our definition (Table 1). A narrow area internarica is also described in other Diprotodontia and Didelphiomorphia. However, in this study, the width-to-depth ratio increases through ontogeny in *Caluromys philander* (from 1.03 to 2.74) and *Monodelphis domestica* (from 1.37 to 3.44), and the area internarica becomes wider. The condition of the area internarica among placentals is diverse in relation to the development of the anterior wall of cupula nasi anterior.

**Table 1:** Measurements of depth and width of the area internarica in mm. Ontogenetic stages increase from left to right: *Monodelphis domestica* (ESUT-Mo), *Caluromys philander* (ESUT-C), *Sminthopsis virginiae* (AMNH SR), *Macropus eugenii* (ESUT-M), *Petaurus breviceps* (LANE-P)

	ESUT--Mo10.5	ESUT--Mo11.5	ESUT--Mo8.5	ESUT--Mo63	ESUT--C6	ESUT--C15	ESUT--C13	ESUT--C25	ESUT--C26	AMNH SR 1A	AMNH SR 2A	AMNH SR 3A	AMNH SR 4A	ESUT--M11	ESUT--M16	ESUT--M28	ESUT--M53	LANE--P82A	LANE--P48
Depth	0.22	0.18	0.28	0.32	0.38	0.30	0.54	0.68	0.80	0.12	0.14	0.14	0.12	0.42	0.50	0.93	1.95	0.39	0.40
Width	0.30	0.42	0.68	1.10	0.39	0.32	0.66	1.36	2.19	0.14	0.14	0.21	0.21	0.48	0.97	1.09	1.92	0.59	0.65
Ratio W:D	1.37	2.33	2.43	3.44	1.03	1.07	1.22	2.00	2.74	1.17	1.00	1.50	1.75	1.15	1.94	1.17	0.99	1.51	1.63

#### Evolution

Area internarica is wide in the mammalian ground pattern. In Macropodidae, Octodontidae, and in a few species, it convergently becomes narrow.

### [15] Area internarica: Depth

#### Definition

The area internarica can be short (0), when the anterior end of the septum nasi reaches up to half of the length of the cupula nasi anterior or more, or deep (1) when the septum nasi does not border the fenestra narina.

#### Discussion

A short area internarica is present in many marsupials. Exceptions are *Macropus eugenii*, *Petaurus breviceps* (Fig. 3c)*,* and *Caluromys philander* [47], whose septum nasi is not involved in the boundaries of the fenestra narina. In *Sminthopsis virginiae*, the depth of the area remains stable through ontogeny, although the head length (HL) grows from 3.5 mm to 11.5 mm (Table 1). In the two *P. breviceps* stages, the depth remains stable, as the crown-rump length increases from 9 mm to 11 mm. In other ontogenetic series the depth increases, as in *M. domestica* (from 0.22 to 0.32 mm) and *C. philander* (from 0.38 mm to 0.80 mm), while the relative increase of crown-rump length is greater, from 10 mm to 63 mm in *M. domestica* and from 6 mm to 26 mm in *C. philander*. In *M. eugenii*, depth is 0.42 mm to 1.95 mm and head length is 11 mm to 53 mm.

In *Atelerix albiventris* and *Echinops telfairi*, the septum nasi reaches above the middle of fenestra narina and borders a large part of it. The same condition is reported for *Hemicentetes semispinosus* [39].

#### Evolution

Ancestrally, the area internarica is deep in Mammalia. It independently becomes shallower in a few species.

### [16] True fenestra narina

#### Definition

The true fenestra narina opens rostrally (0), laterally (1), rostrolaterally (2), ventrally (3), or dorsolaterally (4).

#### Discussion

In most studied pouch young marsupials, except for those of *Perameles* sp. ([40]: plate 3–4), the opening of the fenestra narina is lateral. A rostrolateral opening was examined in the latest stage of *Macropus eugenii* and is mentioned for *Isoodon obesulus* [41].

Among placentals, a rostral opening of the fenestra narina (Fig. 4b), as well as a lateral opening such as in *Atelerix albiventris* (Fig. 3d), is common. The opening of the fenestra narina can also change through ontogeny, such as in *Peromyscus maniculatus* [28], since the anterior wall often develops late or varies in its extent. Only for *Loxodonta africana* [25], a ventral opening ([25]: figure 2) and for *Tachyglossus aculeatus* [20] a dorsolateral opening ([20]: figure 12) are reported.

#### Evolution

The ancestral condition for Mammalia is uncertain. The lateral opening of fenestra narina independently closes in Catarrhini, Octodontidae, and in *Miniopterus schreibersi* [31] and other terminal taxa. Peramelidae, Feliformia, and *Macroscelides proboscelides* [48] independently developed a rostrolateral opening, whereas only *L. africana* [25] shows a ventral opening of fenestra narina. Only the outgroup species has a dorsolateral open fenestra narina.

### [17] Lateral fenestra narina: Orientation

#### Definition

Orientation of the lateral fenestra narina can be ventral (0), dorsal (1), or lateral (2).

#### Discussion

In *Monodelphis domestica* and *Caluromys philander*, the orientation of the fenestra narina changes through ontogeny from an early prominent laterally directed opening to a more ventrolaterally directed one (Fig. 5a, b).

In placentals as in marsupials, the orientation of the lateral fenestra narina is most lateral, whereas dorsal and ventral orientations are less common.

#### Evolution

The ancestral condition for Mammalia is uncertain. Marsupials have a lateral orientation in their ground pattern, although some inter- and intraspecific variation exists in marsupials. A lateral orientation is also present in some placentals, including Strepsirhini and few species from other clades. Species from Afrosoricida and from Rodentia show a ventral orientation of the lateral fenestra narina.

### [18] True fenestra narina: Shape

#### Definition

The shape of the true fenestra narina can be oval (0), round (1), or irregular (2).

#### Discussion

Most of the early pouch young marsupials have a large, wide, and rounded fenestra narina that occupies the complete length and height of the cupula nasi anterior. In ontogeny, the shape changes in most of them. In placentals, shape and size are more variable (Figs. 4, 6, 7).

#### Evolution

In the mammalian ground pattern, fenestra narina has a round appearance. Other shapes are developed independently in different species with greatest diversity among placentals.

### [19] Processus alaris superior

#### Definition

The processus alaris superior can be absent (0) or present (1) (= character 1 of Neto [49]). Synonyms: Cartilago alaris of Fischer [10], processus alaris nasi inferior of Fawcett [31].

#### Discussion

In all marsupials, a processus alaris superior develops shortly after birth or is already well-developed at birth (Fig. 2d).

In most placentals, the process develops before birth (Figs. 7c, 8), as the structures surrounding the nares, but in some early placental stages, such as in *Echinops telfairi*, the process is missing, or still pre-cartilaginous like in *Acomys dimidiatus*. On the contrary, in *Galea musteloides* [49], the process becomes reduced through ontogeny, and in *Castor fiber* [38], the process is completely resorbed in adults. In several rodents, the process is separate from the paries nasi in later stages, as in *Octodon degus* [22], *Sciurus vulgaris* [38], and *Peromyscus maniculatus* [28].

#### Evolution

The processus alaris superior is definitively absent only in *Galea musteloides* [49] and in *Aconaemys fuscus* [22].

### [20] Processus alaris superior: Shape

#### Definition

The processus alaris superior can be curved (0), straight (1), or have straight and curved portions (2).

#### Discussion

**Fig. 10:**
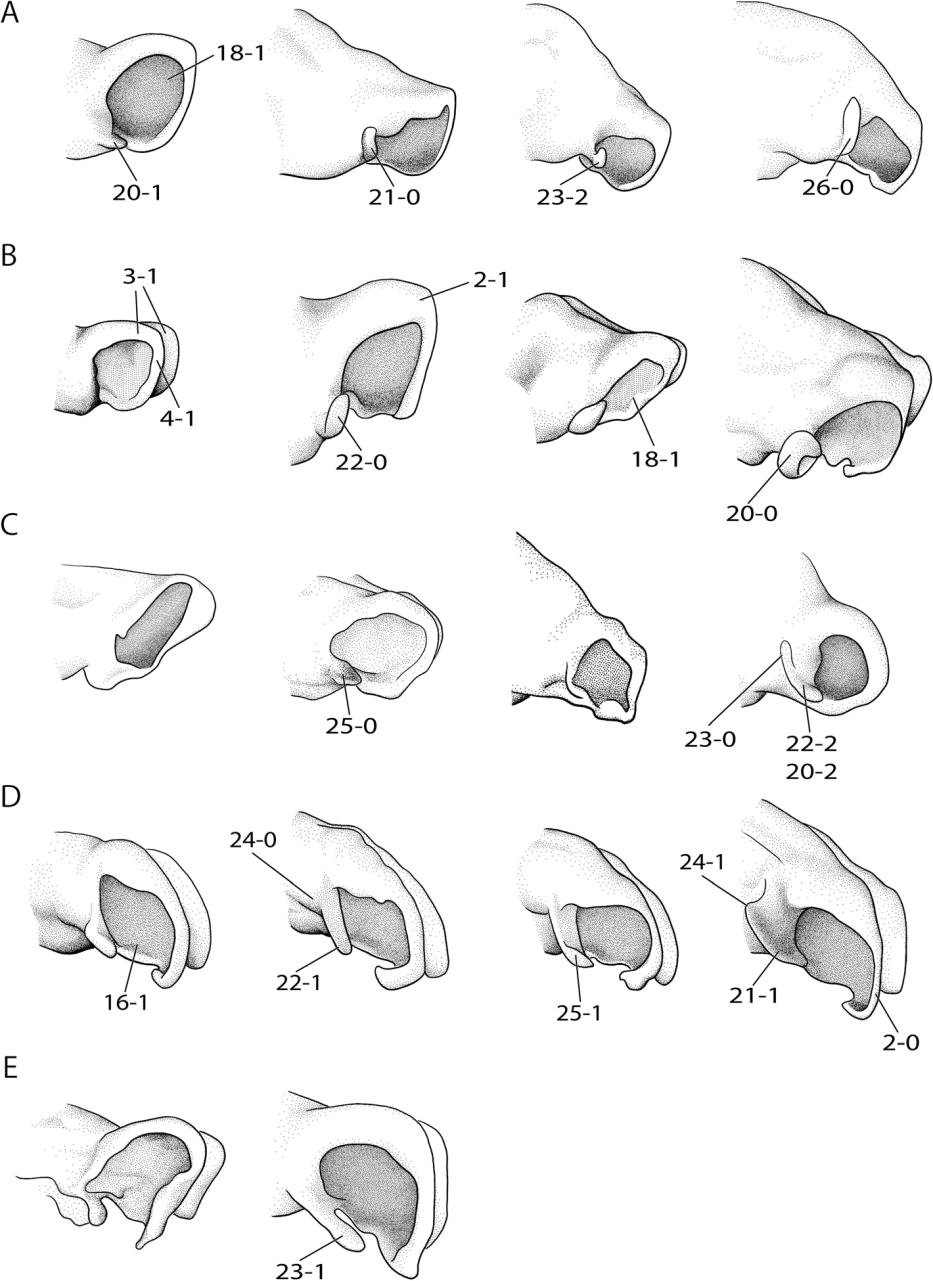
Lateral view of the cupula nasi anterior in ontogenetic series of marsupials. Characters and character states are indicated. Ontogenetic stages increase from left to right. **a** *Monodelphis domestica*: CRL 10.5 mm (ESUT-Mo10.5), 11.5 mm (ESUT-Mo11.5), 20.5 mm (ESUT-Mo8.5) and 63 mm (ESUT-Mo63), **b** *Caluromys philander*: HL 6 mm (ESUT-C6), 7.5 mm (ESUT-C15), 13 mm (ESUT-C13) and 26 mm (ESUT-C26), **c** *Sminthopsis virginiae*: HL 3.5 mm (AMNH SR 1A), 4.5 mm (AMNH SR 2A), 9 mm (AMNH SR 3A) and 11.5 mm (AMNH SR 4A), **d** *Macropus eugenii*: HL 11 mm (ESUT-M11), 16 mm (ESUT-M16), 28 mm (ESUT-M28) and 53 mm (ESUT-M53), **e** *Petaurus breviceps*: CRL 9 mm (LANE-P82A) and 11 mm (LANE-P48). Drawings by Timea Bodogán from 3d-reconstructions. Not to scale

Didelphimorphia (Fig. 10a, b), except for *Didelphis marsupialis* [42], share a pronounced curved laterally-protruding processus alaris superior. The process of *Sminthopsis virginiae* has a straight anterior portion in early stages and later an additional curved lateral one (Fig. 10c). Diprotodontians, in contrast, have a pronounced straight anterior process (Fig. 10d, e). In placentals, as in marsupials, all three conditions occur.

#### Evolution

Ancestrally, the processus alaris superior is straight in Mammalia. It independently becomes curved in all strepsirrhines, *Miniopterus schreibersi* [31], and single species of other clades. Only *Talpa europaea* [6, 10, 50] has straight and curved portions.

### [21] Processus alaris superior: Breadth

#### Definition

The processus alaris superior is narrow (max. half as broad as long) (0) or broad (its breadth is more than half of its length) (1).

#### Discussion

The straight portion is broad in all examined marsupials, and the curved portion is narrow. Whereas in placentals the straight process is narrow in some cases, e.g., in *Octodon degus* [22] and *Sciurus vulgaris* [38], the curved process is broad in others, e.g., in *Miniopterus schreibersi* [31] and *Cryptoprocta ferox* [15]. The relative breadths of the processes do not change through ontogeny.

#### Evolution

The ancestral mammalian condition is uncertain. A narrow processus alaris superior is formed in Didelphimorpha, Scuiromorpha, and in Octodontidae excl. *Octodontomys gliroides* [22]. A broad process is developed in Diprontodonta, Laurasiatheria, and Myomorpha.

### [22] Processus alaris superior: Protrusion

#### Definition

The processus alaris superior protrudes laterally (0), anteriorly (1), or both laterally and anteriorly (2).

#### Discussion

In all examined marsupials, stages with a processus alaris superior that protrudes in the anterior and lateral direction are present. Either the anterior or the lateral part appear first. In placentals, the condition with both an anterior and a lateral protrusion is only reported for *Peromyscus maniculatus* [28].

#### Evolution

The ancestral mammalian condition is uncertain. Theria ancestrally shows an anterior protrusion of the processus alaris superior. *Tachyglossus aculeatus* [20], Soricidae, and Scrotifera develop a lateral protrusion with only *Bos taurus* [6] showing a reversal within the latter taxon. In addition to the anterior orientation, a lateral orientation can be found in *Petaurus breviceps*, *Macropus eugenii*, and *Peromyscus maniculatus* [28].

### [23] Processus alaris superior: Extent

#### Definition

The protruding processus alaris superior can extend dorsally (0), rostrocaudally (1), or both dorsally and rostro-caudally (2) (= character 27 of Frahnert [38] and character 5 of Freyer [36]).

#### Discussion

The direction of the extension of the processus alaris superior changes through ontogeny. In specimens with only a lateral protruding process [see character 22(0)], a rostrocaudal extension of the process appears after the dorsal one. Most placentals have either a dorsally or a rostrocaudally extended process, but not both.

#### Evolution

Ancestrally, the processus alaris superior extends rostrocaudally in Mammalia. Soricidae and Pegasoferae evolved a dorsal extension. Only *Bos taurus* [6] shows a clear dorsal and rostrocaudal orientation of the process.

### [24] Processus alaris superior: Connection

#### Definition

The processus alaris superior can be without (0) or with (1) a portion along the paries nasi.

#### Discussion

In the latest stage of *Macropus eugenii*, the anterior part of the processus alaris superior is extended in the caudolateral direction and is continuously connected to the paries nasi. This condition is unique among marsupials. In *Sminthopsis virginiae*, the process is, like in *M. eugenii*, extended in the caudolateral direction and continuous with the paries nasi, but in contrast to *M. eugenii*, a laterally-curved portion connects to the caudal end of the extension.

Information on this character is missing for most marsupials and placentals in the literature.

#### Evolution

Ancestrally, the processus alaris superior does not develop a portion along the paries nasi in Mammalia. Such a portion develops only in *Sciurus vulgaris* [38] and, as a matter of variation, in two marsupials, i.e. in *Sminthopsis virginiae* and *Macropus eugenii*.

### [25] Processus alaris superior: Size

#### Definition

The length of the processus alaris superior can be small (less than a third of the fenestra narina) (0) or large (about half of its length) (1). In dorsally directed processes, large is when the upturning portion is longer than the horizontal one.

#### Discussion

The earliest specimens of the examined diprotodontians already have a large processus alaris superior. Whereas in didelphids and dasyurids the process starts growing in the earliest stages, it increases in size during ontogeny. Information on this character is missing for most placentals and marsupials.

#### Evolution

The ancestral condition of Mammalia is uncertain. Only in a few species a large process is developed.

### [26] Processus alaris superior: Portion

#### Definition

The processus alaris superior can have one (0) or two parts (1).

#### Discussion

Most marsupials have a processus alaris superior that is an undivided structure. An exception is *Sminthopsis virginiae* with a process with two distinct parts that develop in later ontogeny. In placentals, processes with one or two parts are present. In species with a divided process, one part mostly protrudes in lateral and the other one in anterior direction.

#### Evolution

The ancestral condition of Mammalia is uncertain. *Tachyglossus aculeatus* [20] has two parts of processus alaris superior (Fig. 2a), whereas only one is present in the therian ground pattern. A processus alaris superior with two parts independently evolved in Laurasiatheria, Muroidea, and with ontogenetic variation in *Monodelphis domestica* [36] and *Sminthopsis virginiae*.

### [27] Lamina transversalis anterior

#### Definition

The lamina transversalis anterior can be absent (0) or present (1).

#### Discussion

For marsupials, the development of this structure has been thoroughly documented [51]. In *Echinops telfairi* and *Acomys dimidiatus*, the lamina transversalis develops earlier than elements of the cupula nasi anterior.

#### Evolution

Except for Hominidae ([43, 44]: figure 7) the lamina transversalis anterior is present in all mammals.

### [28] Lamina transversalis anterior: Breadth

#### Definition

The lamina transversalis anterior can be narrow (when the orientation of its median portion is oblique or vertical) (0) or broad (when the orientation of its median portion is horizontal) (1).

#### Discussion

In early stages of marsupials, the lamina transversalis is broad, with its median portion becoming relatively narrow through ontogeny (Fig. 5d). In *Atelerix albiventris,* the lamina transversalis anterior is as broad as the floor of the cupula nasi anterior.

#### Evolution

The ancestral condition of Mammalia is uncertain. The lamina transversalis anterior is broad in Marsupialia except for *Vombatus ursinus* [52]. Ontogenetic variation occurs in *Macropus eugenii* and *Caluromys philander*. Placentalia is characterized by a narrow lamina transversalis anterior. In Eulipotyphla, excl. *Talpa europaea* [6, 10, 50], and in *Castor fiber* [38], it independently became broader.

### [29] Lamina transversalis anterior: Level relative to septum nasi

#### Definition

The level of lamina transversalis anterior can be below (0), at the same level (1), or above (keel) (2) the ventral edge of septum nasi (= character 23 of Frahnert [38]).

#### Discussion

In most marsupials, the lamina transversalis anterior is at the same level as the ventral edge of the septum nasi (Fig. 5c, d). The level is below the septum nasi only in *Caluromys philander*, and above in *Petaurus breviceps*. Among placentals, all three conditions are present.

#### Evolution

Ancestrally in Mammalia, the lamina transversalis anterior is at the same level as the ventral edge of septum nasi. In Hystricomorpha, *Castor fiber* [38], and *Petaurus breviceps*, the lamina is above the ventral edge of septum nasi (keel). A position below the ventral edge of septum nasi is rare among mammals only seen in: *Caluromys philander*, *Setifer setosus* [30], and *Sus scorfa* [53].

### [30] Lamina transversalis anterior: Orientation of rostral portion

#### Definition

The rostral portion of the lamina transversalis anterior can be oblique (0), horizontal (1), or have a vertical portion (2).

#### Discussion

The rostral most region of the lamina transversalis anterior is variable in shape and its orientation changes through ontogeny. In the ontogenetic series of didelphids and *Sminthopsis virginiae*, the lamina rostrally forms a horizontal plane that increases in convexity in the caudal direction, until it separates from the septum nasi in the region where the ductus nasopalatinus opens into the nasal cavity. In *Macropus eugenii*, a major change in the orientation of the lamina occurs as well. Whereas in the earliest stage the anterior portion of the lamina is an oblique (Fig. 5c), mediolaterally-oriented plane, its steepness increases with proceeding development. In later stages, the medial horizontal section is reduced, while the lateral vertical section participates in the formation of the sidewall of the nasal capsule (Fig. 5d), as reported for monotremes [20] and some placentals. For placentals, all conditions are reported.

#### Evolution

The ancestral condition of Mammalia is uncertain. Whereas the rostral portion of the lamina transversalis anterior has a vertical portion in *Tachyglossus aculeatus* [20], it is horizontal in the therian ground pattern. It becomes independently oblique in Diprontodontia, *Setifer setosus* [30], Rodentia, Lemuriformes, and *Miniopterus schreibersi* [31]. Some variation in the orientation of the lamina transversalis anterior exists in different species among Theria.

### [31] Lamina transversalis anterior: Length

#### Definition

The lamina transversalis anterior can be short (i.e., shorter than the cupula nasi anterior) (0) or long (i.e., the lamina is longer than the cupula nasi anterior) (1).

#### Discussion

The length of the lamina transversalis anterior increases in all examined marsupials through ontogeny. In contrast to *Macropus eugenii*, *Petaurus breviceps* has a very short lamina transversalis anterior and, additionally, in its earliest stage, the caudo-lateral margin of the lamina that is continuous with the paries nasi is elongated in the ventral direction forming a lamina infraconchalis as was described for *Wallabia rufogrisea* [54] and *Didelphis marsupials* [42] and was depicted for *Perameles* sp. [40]. This condition was not observed in the other specimens of our sample. Long ([6]: plate II) and short ([32, 33]: figure 7) lamina transversalis anterior occur in placentals.

#### Evolution

Ancestrally, mammals have a short lamina transversalis anterior. Independently, it becomes long in six marsupials and four placental species.

### [32] Zona annularis

#### Definition

The zona annularis can be absent (0) or present (1) (= character 13 of Freyer [36]).

#### Discussion

Most of the examined marsupial specimens have a rather short zona annularis. However, in none of the examined ontogenetic series, the zona annularis is completely closed, and it can be missing in early or late stages. A zona annularis is reported for most marsupials (Fig. 1e).

*Atelerix albiventris* and *Echinops telfairi* have a zona annularis like most of the placentals reported in the literature. A missing zona annularis is reported for Hominidae [7, 26], including *Homo* [43, 44].

#### Evolution

Zona annularis is present in the ground pattern of Mammalia. It is lost in some marsupials, *Vombatus ursinus* [52] and *Trichosurus vulpecula* [36], and occurs with ontogenetic variation in *Monodelphis domestica* and *Macropus eugenii*. Among placentals, it is lost in Lagomorpha, *Miniopterus schreibersi* [31], *Galago senegalensis* [55], and *Castor fiber* [38].

### [33] Zona annularis: Connection with septum nasi

#### Definition

Lamina transversalis anterior is either fused (1) or not fused (0) with the septum nasi (1) (= character 10 of Frahnert [38]).

#### Discussion

The lamina transversalis anterior and the septum are only separated by a very thin fissure on one side in an early stage of *Monodelphis domestica.* In all other marsupials, the septum and the lamina are continuously connected (Fig. 5c). In many placentals, however, the septum nasi and the lamina transversalis anterior are disconnected.

#### Evolution

In the mammalian ground pattern, the lamina transversalis anterior is fused with the septum nasi. It is not fused in Lagomorpha and in several other placental species, including *Miniopterus schreibersi* [31], some carnivorans, some primates, and others. In marsupials, the mammalian ground pattern is preserved.

### [34] Zona annularis: Connection with paries nasi

#### Definition

The lamina transversalis anterior is either fused (1) or not fused (0) with the paries nasi (1).

#### Discussion

In most marsupials the paries nasi is fused with the lamina transversalis anterior (Fig. 5c). The resorption of the paries nasi in the late stage of *Macropus eugenii* leads to a disconnection from the lamina transversalis anterior (Fig. 5d). The only other marsupials for which a disconnection between paries nasi and lamina transversalis anterior is reported is *Vombatus ursinus* [52]. In placentals, a connection between paries nasi and lamina transversalis anterior is almost always present.

#### Evolution

Except for *Vombatus ursinus* [52], *Macropus eugenii*, and *Neomys fodiens* [56], all mammals have the lamina transversalis anterior fused with the paries nasi.

### [35] Fenestra internasalis anterior

#### Definition

The fenestra internasalis anterior can be absent (0) or present (1) (= character 21 of Frahnert [38] and character 11 of Freyer [36]). Synonyms: Fenestra septi nasi of Gaupp [29], fenestra lateralis of Reinbach [32, 33].

#### Discussion

A fenestra internasalis anterior is missing in all marsupials examined so far (Fig. 3c) ([19], this study).

Likewise, in many placentals the fenestra internasalis anterior is missing. In *Atelerix albiventris* (Fig. 3d) and *Erinaceus europaeus* [6], the septum nasi is fenestrated posteriorly to the lamina transversalis anterior. In *Acomys dimidiatus*, the septum is fenestrated in the region of the cupula nasi anterior. In some species, the fenestra internasalis is not present in every stage, as in the ontogenetic series of *Peromyscus maniculatus* [28].

#### Evolution

Ancestrally, Mammalia developed a fenestra internasalis anterior. It is absent in Marsupialia, in *Procavia capensis* and *Loxodonta africana* [25], in Afrosoricida, Cetartiodactyla, Carnivora, *Suncus orangiae* [30], *Castor fiber* [38], *Petromus typicus* [18], and *Phodopus sungorus* [46].

### [36] Fenestra superior nasi

#### Definition

The fenestra superior nasi can be absent (0) or present (1) (= character 2 of Freyer [36]). Synonym: Fenestra dorsalis of Fawcett [57].

#### Discussion

A fenestra superior nasi is missing in all marsupials. In placentals, a fenestrated tectum nasi in the anterior region of the nasal capsule is common (Figs. 4a, b, d, 6a, 7a, 8a). In *Atelerix albiventris*, the reason for the missing fenestra might be a not yet fully chondrified cupula nasi anterior in our sampling.

#### Evolution

The ancestral condition of Mammalia is uncertain. Fenestra superior nasi is absent in all marsupials. Among placentals, it is present in Atlantogenata, Eulipotyphla with the exclusion of *Atelerix albiventris*, Scandentia, Muroidea, Lagomorpha, and few distantly related species.

### [37] Sulcus supraseptalis

#### Definition

The sulcus supraseptalis can be absent (0) or present (1).

#### Discussion

The sulcus supraseptalis is present in all marsupials except in the earliest stages of *Caluromys philander* and *Sminthopsis virginiae*. Likewise, most placentals have a sulcus supraseptalis (Fig. 6a).

#### Evolution

Sulcus supraseptalis is present in the mammalian ground pattern and was only lost in *Jaculus jaculus* and *Acomys* sp. [28].

### [38] Sulcus supraseptalis: Depth

#### Definition

In the anterior region of the lamina transversalis anterior the sulcus supraseptalis can be shallow (tectum nasi is flat above the nasal cavities) (0) or deep (tectum nasi arches above the nasal cavities) (1).

#### Discussion

In *Sminthopsis virginiae* and *Monodelphis domestica*, the depth of the sulcus increases through ontogeny, whereas in *Caluromys philander* the depth of the sulcus varies in ontogeny and is shallow again at a late stage. In the ontogenetic series of *Macropus eugenii*, the sulsuc supraseptalis remains deep (Fig. 5c, d).

#### Evolution

Ancestrally in Mammalia, the sulcus supraseptalis is deep. In three marsupials, we documented ontogenetic variation. Among placentals, Myomorpha and three distantly related species have a shallow sulcus supraseptalis.

### [39] Marginoturbinale

#### Definition

The marginoturbinale can be absent (0) or present (1) (= character 26 of Freyer [36]).

#### Discussion

Most marsupials have a marginoturbinale in the earliest stages (Fig. 1d). In all placentals, the marginoturbinale is present.

#### Evolution

The marginoturbinale is present in the mammalian ground pattern. It is lost in some marsupials, including *Didelphis marsupialis*, *Isoodon obesulus* [41], *Sminthopsis virginiae*.

### [40] Atrioturbinale

#### Definition

The atrioturbinale can be absent (0) or present (1) (= character 27 of Freyer [36]).

#### Discussion

Except for some early stages of marsupials and the monotreme *Tachyglossus aculeatus* [20], all mammals have an atrioturbinale (Fig. 1e).

#### Evolution

The ancestral condition of Mammalia is uncertain. Whereas the outgroup species *Tachyglossus aculeatus* [20] has no atrioturbinale, it is present in Theria with few exceptions among marsupials.

### [41] Maxilloturbinale

#### Definition

The maxilloturbinale can be absent (0) or present (1) (= character 30 of Freyer [36]).

#### Discussion

In early ontogenetic stages of marsupials, the maxilloturbinale can be missing as in *Isoodon obesulus* [41] and likely in *Petaurus breviceps*, or is not pronounced, whereas in late stages all marsupials have a well-developed maxilloturbinale (Fig. 3b). In all placentals, the maxilloturbinale is present.

#### Evolution

Except for *Isoodon obesulus* [41] and likely for *Petaurus breviceps* among marsupials, the maxilloturbinale is present in early ontogenetic stages of all mammals.

### [42] Steepness of ductus nasopalatinus

#### Definition

The connection of the oral and nasal cavity by the ductus nasopalatinus can be shallow (in cross section the ductus nasopalatinus is visible as a circle or a slit only open to one cavity) (0) or steep (in cross section the ductus nasopalatinus is visible as vertical connection between the two cavities) (1).

#### Discussion

In all early marsupial stages examined, the oral and nasal cavity are connected by a horizontal ductus nasopalatinus (Fig. 3a), whereas in later stages the ductus nasopalatinus is vertically oriented (Fig. 3b). For marsupials and placentals from literature, no information is available.

#### Evolution

Due to limited data, which only show ontogenetic variation among some marsupial species, we cannot reconstruct the evolution of this character.

### [43] Commissura alicupularis

#### Definition

The commissura alicupularis can be absent (0) or present (1) (= character 6 of [36]). Synonyms: Ali-cupular commissure of Fawcett [31], anulus alaris of Gaupp [29].

#### Discussion

In none of the examined marsupial specimens, a commissura alicupularis is present, and the condition is not described for other marsupials. In the late stages of *Sminthopsis virginiae*, the processus alaris superior and cartilago cupualaris approach each other.

In placentals, a commissura alicupularis is only reported for *Hemicentetes semispinosus* [39], *Procavia capensis* ([25]: figure 3), *Miniopterus schreibersi* ([31]: figure 20), and *Sus scrofa* ([53]: plate XXXIV, figure I).

#### Evolution

The commissura alicupularis is absent in all mammals, except for Afrotheria and Scrotifera.

## Discussion

There is much ontogenetic and phylogenetic diversity of the anterior part of the nose capsule in mammals. We were able to discriminate characteristics that are variable through early ontogeny and characters that are more stable within a species and hence more useful for phylogenetic comparisons. A character mapping revealed a number of derived characters for particular clades within Mammalia.

### Ontogenetic changes of characters

Among the marsupials studied, we found ontogenetic variation for almost 50% of the defined characters. Our results will be valuable to (I) study character transformation in particular species, (II) test in ontogenetic series whether the apomorphies detected herein actually represent derived characters in evolution or just an artifact of ontogenetic variation, and (III) test for homologies.

Addressing the challenges of comparing chondrocranial anatomy among species, Werneburg and Yaryhin [9] defined a ‘tempus optimum’ stage for comparisons based on defined parameters. In their example, using reptile chondrocrania, they defined the start of tempus optimum when the ethmoid region is fully developed, because chondrification starts posterior in the skull and develops anteriorly [20, 30]. The endpoint of development was defined when the basicranium starts to ossify because then the chondrocranium is resorbed or ossifies drastically.

Structures of the cupula nasi are assumed to be the last ones of the nasal capsule to chondrify [30], whereas the septum nasi is the first to do so [30, 50]. In many placentals, the cupula nasi only develops when other parts of the nasal capsule are already resorbed and ossification of cranial bones has started [24, 30]. This is in contrast to the case of marsupials, in which resorption of the nasal cartilage starts after birth, whereas ossification of the premaxilla starts around birth [58]. Nevertheless, we suppose our coding is a good representation of interspecific variation among placentals, but agree that uncertainties could be involved based on limited data availability.

Nonetheless, many characters do not show ontogenetic variation in their presence in the marsupial species studied herein. We consider these as informative characters in a phylogenetic sense despite the ontogenetic progress of their development.

### Marsupialia

After birth, marsupials attach to the teats of the mother and keep fixed to it for a long period [21, 59, 60]. In that process, the skin of the mother and the snout of the young partly fuse. Marsupial newborns are reported to breathe through the skin in early postnatal life [61–63]. However, the respiratory system is also well developed for simultaneous sucking and breathing [64].

Marsupials, at the time of birth, are conservative in the shape of the external nares. The nares are of large size and round shape, with nasal swellings surrounding them. Distinct organs of the anterior nose region are not yet present; the specific structures of the rhinarium develop later ([65], this study).

Marsupials have a large cartilago cupularis with a distinct area internarica in the ground pattern. The developed anterior wall leads to a lateral opening of the true fenestra narina, although some inter- and intraspecific variation exists. The lamina transversalis anterior of marsupials is broad and fused with the septum nasi. A fenestra internasalis anterior and a fenestra superior nasi, which would reduce stability, are absent.

The cupula nasi of early pouch young marsupials is simple in shape and is surprisingly uniform across the group. Processes, to which facial muscles attach and which support the soft tissue structures surrounding the nares, are in most cases rudimentary or not present in early ontogeny. Facial muscles and the other soft tissue only start developing after birth [36, 66]. A stable rostral most portion of the nasal capsule corresponds to functional demands in early postnatal life. The lateral fenestra narina probably facilitates respiration [36] when pouch young are permanently attached to the mother’s teat in their first weeks of life. The broad lamina transversalis anterior supports the stability to withstand the biomechanical impact on the cupula nasi anterior while attached to the teat [20]. All these characters, in sum, indicate the importance of a stable cupula nasi anterior in early pouch young.

### Placentalia

Placentals are characterized by various reductions of the cupula nasi anterior, with the opening of the true fenestra narina and the presence of an area internarica, depending on the development of the anterior wall. Ventrally situated elements of the cartilago cupularis can be reduced or missing, while the dorsal lateral lamina increases in size ([11], this study). The lamina transversalis anterior of placentals is narrow. A fenestra internasalis anterior and a fenestra superior nasi are common. A commissura alicupularis is present in few placental species only.

Many characters indicate a mobile fetal cupula nasi [39]. The variations in the opening of the true fenestra narina and the processes of cupula nasi anterior are reflected in the appearance of the external nares at birth. The shape and position of the external nares in neonate placentals is more variable than in marsupials and resembles their adult shape. In contrast to marsupials, muscles and ligaments, related to mobility of the cupula nasi and the external nares, attach to different structures of the cupula nasi anterior already at birth [22, 30, 41, 67].

### Theria

The hypothetical therian ancestor was characterized by a cupula nasi anterior with a complete floor, a wide and deep area internarica, and a cartilago cupularis with an anterior wall. The cartilago cupularis lacked a processus cupularis. The lateral laminae of the cartilago cupularis were longer than its medial lamina. The dorsolateral laminae of the cartilago cupularis had at least the same length as the ventrolateral lamina. The processus lateralis ventralis had half of the breadth of the cartilago cupularis and was continuously connected with the lamina transversalis anterior. A lamina transversalis anterior with a zona annularis was present and was placed at the same level as the ventral edge of septum nasi. The septum nasi was fenestrated. The processus alaris superior was straight and rostrocaudally extended.

It has been hypothesized that a complete cupula nasi anterior [36] and a broad rhinarium [68] were part of the therian ground pattern (i.e., characterized the last common ancestor of the group). In fact, almost all therian mammals have a cupula nasi anterior; major reductions of the cartilago cupularis occurred only within the evolution of placentals ([11, 12, 14, 18, 21, 24, 46], this study). We reconstructed the therian ancestor as it having had an anterior wall and a wide area internarica. Although orientation of the fenestra narina in Theria was uncertain in our analysis, the presence of an anterior wall implicates a lateral opening of the fenestra narina [26]. Rostrolateral and lateral oriented fenestra narina are also known for many placentals. The condition is not necessarily associated to a fixation to the teat. The orientation of the lamina transversalis anterior in the ancestral condition of Mammalia is uncertain, whereas in the therian ground pattern the orientation is horizontal. In phylogeny, it primarily was an element of the floor [20]. The presence of a fenestra internasalis anterior was hypothesized to be a derived condition in placentals [39]. This is in contrast to our study, where it is reconstructed as being present in the therian ground pattern.

Although many characteristics of the anterior nose region of marsupials and placentals were not developed yet in the therian ancestor, our reconstruction shows that the latter did not show any structural nasal fixation similar to that of marsupials. The therian ancestor likely had an ethmoid region more similar to placentals in functional regards. That means that the anterior nasal region of early therians was structurally more labile than in marsupials, indicating that the newborns of the therian ancestor were not fixed to the mother’s teat. This confirms recent reconstructions of Werneburg et al. [69], who found that at birth the therian ancestor was more placental-like in having well-developed fore- and hind limbs.

### Functional considerations

For *Oryctolagus cuniculus*, Voit [8] speculated that the reduction of the elements of the cupula nasi and the solum nasi is related to the use of the rostral end of the snout for tactile exploration, whereas Schunke and Zeller [39] correlated a high mobility of the snout in *Hemicentetes semispinosus* to the shape of the cupula nasi. In aquatic mammals [11, 14] and primates [24], the reduction of the cupula nasi is reportedly related to the reduction of the sense of smell.

A reduced cupula nasi is not known in marsupials. In newborns, the development of the olfactory system varies in different species [70–72], reflecting differing developmental maturity at birth [70]. Studies indicate that in some neonate marsupials the olfactory system is capable to detect olfactory cues [71–73]. A well-developed cupula nasi at birth may provide stability in this rostral-most part of the chondrocranium (see above, [19]). Mess [12] mentioned that a discontinuous rostral nasal floor in hystricognath rodents indicates, as in lagomorphs, a functional shift to a more mobile cupula nasi anterior. Voit [8] speculated that in *Talpa europaea* the well-developed cupula nasi is related to mechanical stress to the rostrum related to digging.

## Conclusion

1. We found that marsupials at birth are characterized by a complete but simple and robust cupula nasi anterior, whereas the elements of the cupula nasi vary in their presence and complexity in placentals around birth. The robust cupula nasi in marsupials, and the more mobile one in placentals, are probably related to functional requirements after birth.
2. In the reconstructed therian ancestor, the conditions of characteristics typical of marsupials, such as a large cartilago cupularis, a broad lamina transversalis anterior, and the lateral opening of the fenestra narina cannot be reconstructed with certainty. Current data, however, lead to the hypothesis that the common ancestor of placentals and marsupials was placental-like.
3. The pouch young series of marsupials allowed us to trace characters through ontogeny.
4. Besides the differences in shape, placentals and marsupials differ in the period of the development of the cupula nasi. In placentals, the time is more variable, coupled with the longer gestation typical of this group. Therefore, the cupula nasi anterior is developed in fetal stages, and ossification in the rostral region of the skull, as resorption of the cartilage of the nasal capsule, also start around birth. In contrast, in marsupials the cupula nasi is just developed at the time of birth. Ossification starts shortly before birth, but resorption occurs only after birth.
5. Individual variation in development of the anterior portion of the chondrocranium is rarely studied but needs to be investigated as it is of potential significance to understand character identity. Finally, new imaging techniques such as micro computed tomography with soft tissue staining [74] could help to increase the number of examined specimens and species.
6. There are several derived characters for major mammalian taxa within Marsupialia and Placentalia, which may be casually related to changes in lifestyle and developmental constraints. As the characters defined herein are very specific, clear correspondences to functional specializations are not easy to detect. Due to ontogenetic changes of particular characters, we avoided over-interpreting these patterns. Nonetheless, we can support with confidence that chondrocranial anatomy shows strong correspondence to functional anatomy and phylogeny.

## Methods

**Fig. 11:**
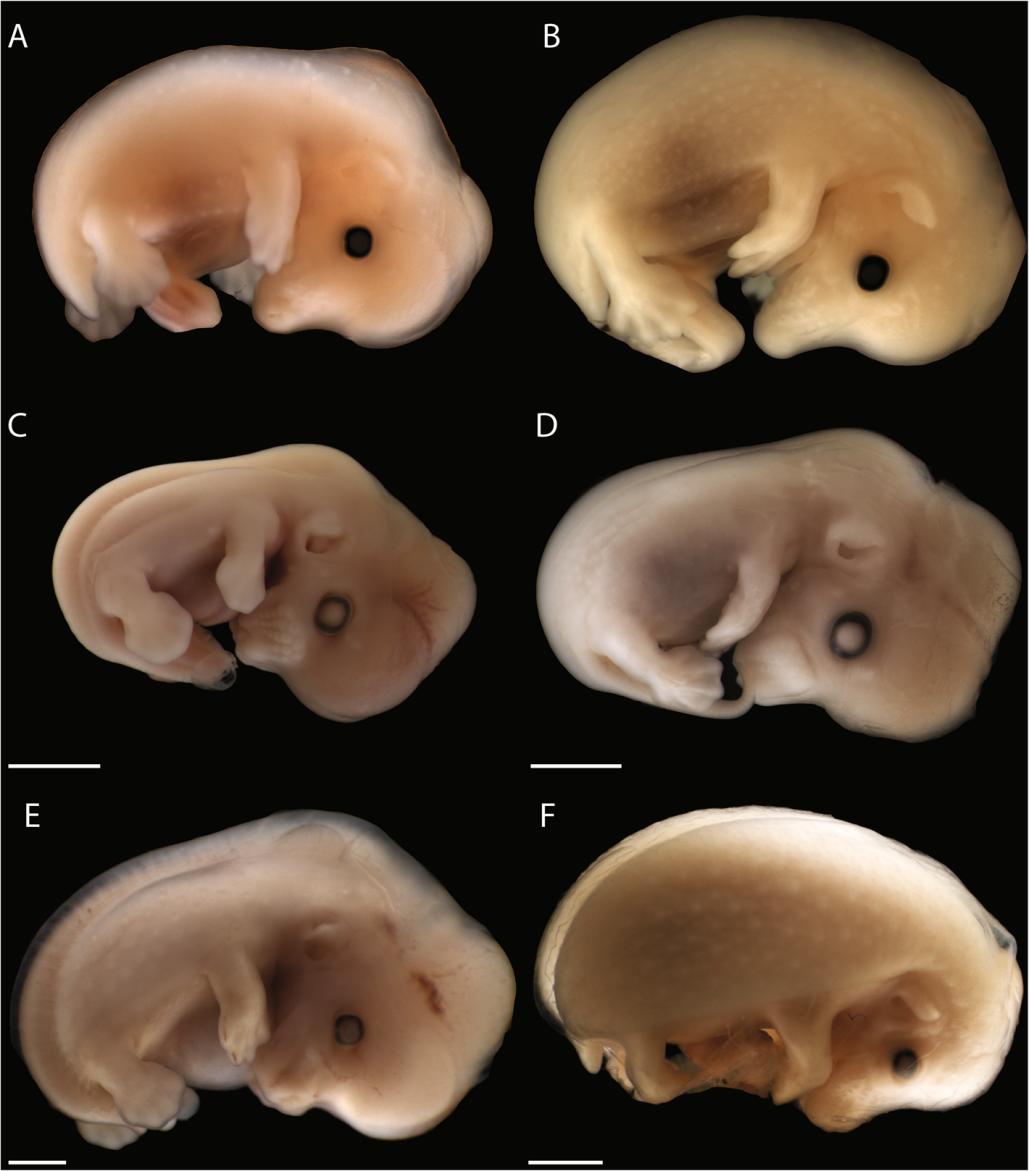
Embryonic stages of placentals. **a** *Echinops telfairi* (LANE-Ech5a) (mirrored), **b** *Echinops telfairi* (LANE-Ech7a) (mirrored). **c** *Acomys dimidiatus* (LANE-Aco18, dpc 18), **d** *Acomys dimidiatus* (LANE-Aco21, dpc 21), **d** *Atelerix albiventris* (LANE-Atx21, dpc 21), **d** *Atelerix albiventris* (LANE-Atx25, dpc 25). Scale bars equal 1 mm, *Echinops telfairi* (A-B) not to scale

Serial histological sections of chondrocrania of three placental (Fig. 11a–f) and five marsupial species were examined (Table 2). The three-dimensional structure of the anterior part of the cartilaginous nasal capsule of embryos, neonates, and pouch young was reconstructed.

**Table 2:** Specimens list: histological sections

Systematics	Species	Specimen	HL [mm]	CRL [mm]	age [dpn]	Collection number
Didelphimorphia	*Monodelphis domestica*	K. Smith’s Colony, Duke University	ca. 4.5	10	0	ESUT-Mo4.5
			6	10.5	2	ESUT-Mo10.5
				11.5	5	ESUT-Mo11.5
				16	9	ESUT-Mo16
		K. Smith’s Colony, Duke University	8.5	20.5	12	ESUT-Mo8.5
				63	40	ESUT-Mo63
	*Caluromys philander*	C.1 (ex. Coll. Charles-Dominique) Paris	6	11.5	PY [pouch young]	ESUT-C6
		C.2 (ex. Coll. Charles-Dominique) Paris	7.5	15	PY	ESUT-C15
		Collection of M. Sánchez	13		30	ESUT-C13
		Collection of M. Sánchez	25		77	ESUT-C25
		Collection of M. Sánchez	26		84	ESUT-C26
Dasyuromorphia	*Sminthopsis virginiae rufigenis*	AMNH SR 1A	3.5	5	PY	AMNH SR 1A
		AMNH SR 2A	4.5	7.5	PY	AMNH SR 2A
		AMNH SR 3A	9	14.5	PY	AMNH SR 3A
		AMNH SR 4A	11.5	18.5	PY	AMNH SR 4A
Diprotodontia	*Macropus eugenii*		11		PY	ESUT-M11
			16		PY	ESUT-M16
			28		PY	ESUT-M28
			53		PY	ESUT-M53
	*Petaurus breviceps*	82A		9	PY	LANE-P82A
		48	6 mm	11	PY	LANE-P48
					age [dpc]	
Afrosoricida	*Echinops telfairi*	5a (Collection of M.C.M.)			embryo	LANE-Ech5a
		7a (Collection of M.C.M.)			embryo	LANE-Ech7a
Rodentia	*Acomys dimidiatus*	E18 (Collection of M.C.M.)			18	LANE-Aco18
		E21 (Collection of M.C.M.)			21	LANE-Aco21
Eulipotyphla	*Atelerix albiventris*	E21 (Collection of M.C.M.)			21	LANE-Atx21
		E25 (Collection of M.C.M.)			25	LANE-Atx25

### Specimens

**Fig. 12:**
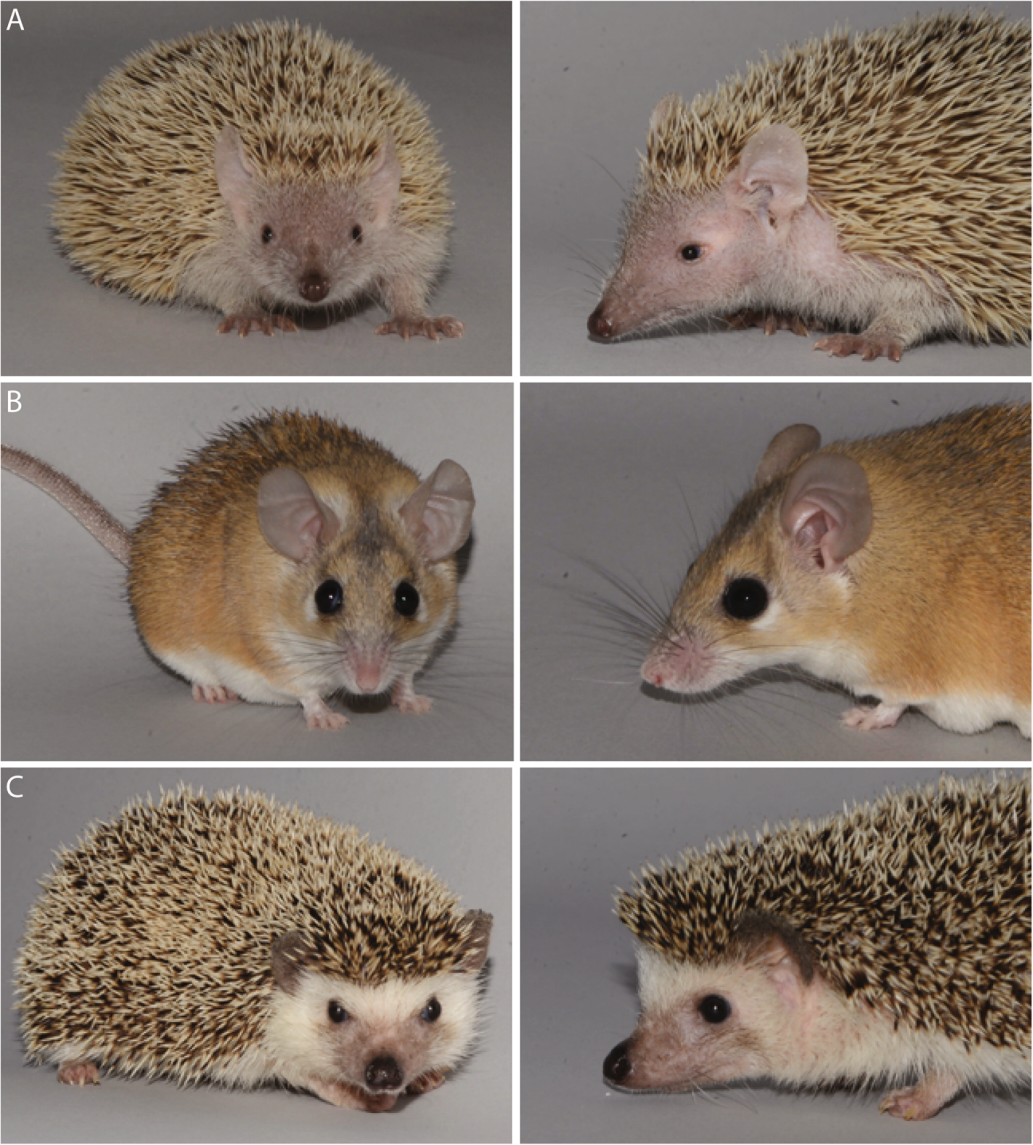
Frontal view of adult placentals on the left, lateral view on the right. **a** *Echinops telfairi*, **b** *Acomys dimidiatus*, **c** *Atelerix albiventris*

Histological sections of *Macropus eugenii*, the tammar wallaby, are housed at Embryologische Sammlung, Universität Tübingen. The histological sections of *Monodelphis domestica*, the grey short-tailed opossum, *Caluromys philander*, the bare-tailed woolly opossum, and *Sminthopsis virginiae*, the red-cheeked dunnart, are currently housed at the Evolutionary Morphology and Palaeobiology group, Paläontologisches Institut und Museum der Universität Zürich. The histological sections of *Petaurus breviceps*, the sugar glider, *Echinops telfairi* (Fig. 12a), the lesser hedgehog tenrec, *Acomys dimidiatus* (Fig. 12b), the eastern spiny mouse, and *Atelerix albiventris* (Fig. 12c), the four-toed hedgehog, were produced in and are stored at the Laboratory of Artificial and Natural Evolution, Department of Genetics and Evolution, University of Geneva.

### Histology and 3d-reconstructions

Following standard procedures Mulisch and Welsch [75], all 27 specimens (Table 2) were embedded in paraffin and stained with Azan after Haidenhain or Azan-Domagk, except for the specimens from the LANE, which were stained with Alcian Blue, Hematoxylin, and Orange G. Staining results in orange to red coloration for bones, blue for cartilage, and red to pink for soft tissue.

To identify the sections, they were named by the number of the section on the slide, the column, and the row, with “2–3-5”, for example, meaning slide number two, column three, and section five, or with “23–5” for the sections from LANE, meaning slide number 23, and section five. The opening of ductus nasopalatinus to the oral and to the nasal cavity was used, in most models (see below), as the posterior-most point of the three-dimensional reconstructions.

Every second section was photographed under a Leica DM2500M microscope with a Leica DFC 420C camera in Zürich and a Canon EOS 600D in Tübingen. The last photographed section was the section in which the ductus nasopalatinus was completely open to the nasal cavity. If that section was not available, the section with the complete opening to the oral cavity was used. All sections of the LANE samples were scanned with a Pannoramic MIDI slide scanner, and every second section, containing nasal cartilage, was photographed with Pannoramic Viewer.

The registration of the digitized sections was performed using Adobe Photoshop CS5. The images were aligned manually to a stack, using the most posterior, and therefore the largest section as reference image. The digitized section next to it was loaded on the top, made 50% transparent, and rotated to fit the anatomical structure of the reference image. Several anatomical structures were used as reference for the proper alignment of the sections, because no guiding mark was available. This procedure was repeated with all the digitized sections, and finally the produced layers were exported as aligned image files.

The segmentation and volume rendering were performed using VG Studio Max 2.2. To import the image stack, the voxel size was calculated from the resolution, the scale, and the thickness of the digitized sections. The x- and y-dimensions were calculated from the number of pixels per millimeter, and the thickness of the sections determined the z-dimension. The cartilaginous structure of the nasal capsule on each layer of the image stack was manually segmented with the adaptive polygon tool, and each segmented layer was saved as a new region. All regions were merged, after the distorted sections were excluded, and the three-dimensional surface was extracted as an STL-file. Based on the 3d-reconstruction, we measured depth and width of area internarica and calculated the ratio of both (Table 2).

### Terminology

We use the term ‘cross’ for the plane that divides the head in an anterior and a posterior section, and the term ‘horizontal’ (‘transverse’ in the literature) for the plane that divides the head in a ventral and a dorsal section. The anatomical terminology to describe the cartilaginous structures of the cupula nasi anterior and the nasal capsule follow Maier [56], Mess [22], and Ruf [28].

### Institutional abbreviations

AMNH = American Museum of Natural History, Department of Mammalogy, New York, USA; LANE = Laboratory of Artificial and Natural Evolution, University of Geneva, Switzerland; ESUT = Institut für Evolution und Ökologie, Vergleichende Zoologie, Embryologische Sammlung Universität Tübingen, Germany.

### Phylogenetic analyses

**Fig. 13:**
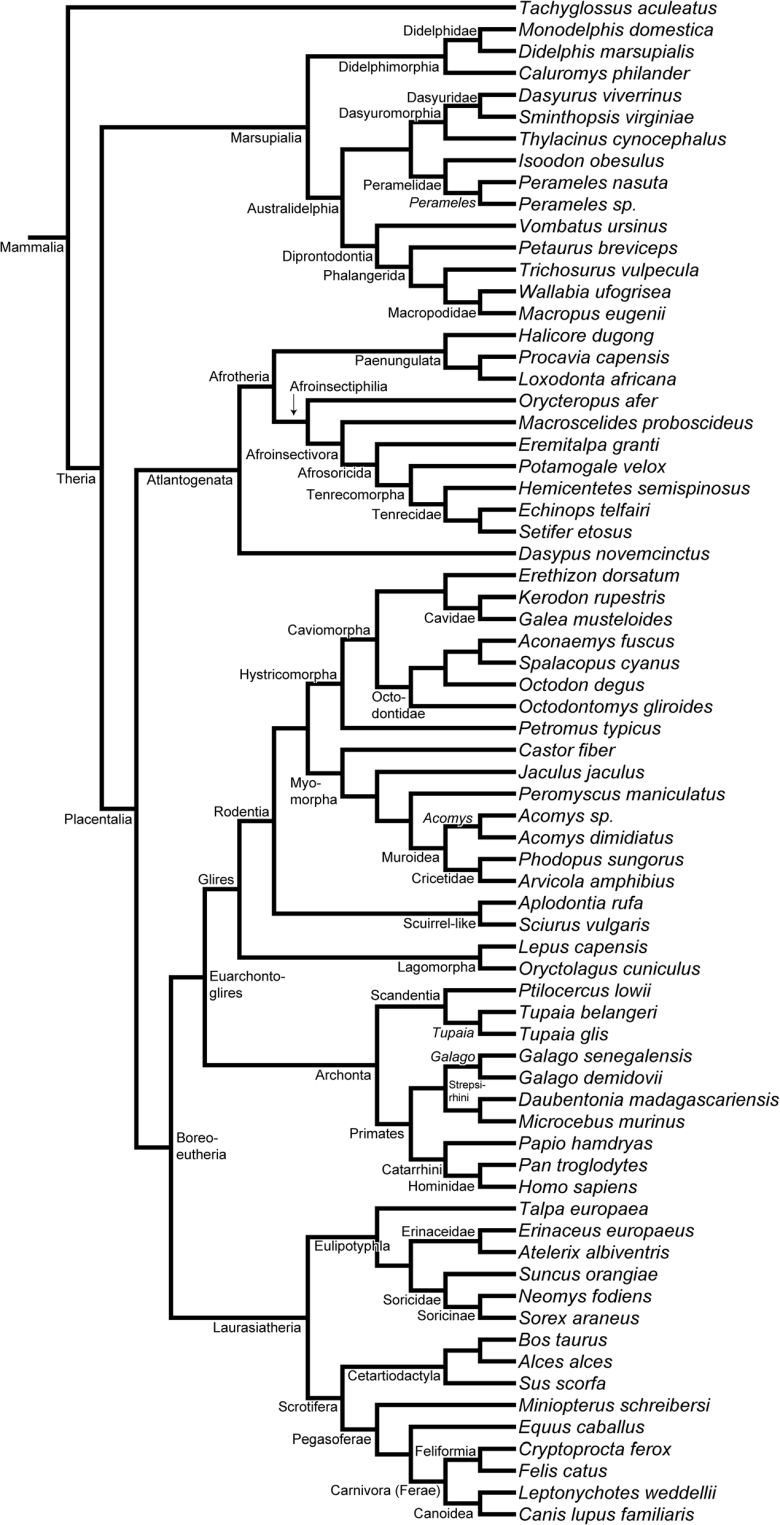
Phylogenetic framework and taxonomic sampling for the character mapping performed in this study. For details on phylogenetic arrangement, see text

For character analysis (Tables 2, 3), we used a topology (Fig. 13) based on Hedges and Kumar [76] for global mammalian phylogeny, with Atlantogenata opposing Boreoeutheria inside Placentalia (see also Foley et al. [77]). On lower taxonomic levels, we relied on Kuntner et al. [78] for Afrotheria, Everson et al. [79] for Tenrecidae, and Opazo [80] for Hystricognathi. Species were used as terminal taxa. In species for which ontogenetic variation was documented by us or in the literature (Appendices 1–2), polymorphism was defined. In order to detect apomorphic character states for particular clades, character mapping was performed in TNT [81]. For that, a tree file was imported from Mesquite 3.40 [82].

**Table 3:** Specimens from literature used for comparison of the cupula nasi anterior

Major taxon	Species	References	HL [mm]	CRL [mm]	Developmental stage	Age/Stage
Monotremata	*Tachyglossus aculeatus*	Kuhn 1971	27	53	pouch young	
	*Tachyglossus aculeatus*	Kuhn 1971		196	pouch young	5–6 week old
	*Tachyglossus aculeatus*	Kuhn 1971	72	182	pouch young	3 month
	*Tachyglossus aculeatus*	Kuhn 1971			adult	
	*Tachyglossus aculeatus*	Kuhn 1971		250	juvenil	
	*Tachyglossus aculeatus*	Kuhn 1971			adult	
	*Tachyglossus aculeatus*	Kuhn 1971			adult	
	*Tachyglossus aculeatus*	Kuhn 1971				
Didelphiomorphia	*Monodelphis domestica*	Freyer 1999			embryo	dpc 13
	*Monodelphis domestica*	Freyer 1999			embryo	dpc 14
	*Monodelphis domestica*	Freyer 1999			neonate	
	*Monodelphis domestica*	Freyer 1999			neonate	
	*Monodelphis domestica*	Freyer 1999			pouch young	dpn 1
	*Monodelphis domestica*	Freyer 1999			pouch young	dpn 5
	*Monodelphis domestica*	Freyer 1999			pouch young	dpn 8
	*Monodelphis domestica*	Freyer 1999			pouch young	dpn 14
	*Monodelphis domestica*	Freyer 1999			pouch young	dpn 15
	*Monodelphis domestica*	Freyer 1999			pouch young	dpn 19
	*Monodelphis domestica*	Freyer 1999			pouch young	dpn 21
	*Monodelphis domestica*	Freyer 1999			pouch young	dpn 25
	*Monodelphis domestica*	Freyer 1999			pouch young	dpn 28
	*Monodelphis domestica*	Freyer 1999			pouch young	dpn 33
	*Monodelphis domestica*	Freyer 1999			adult	
	*Monodelphis domestica*	Freyer 1999			adult	
	*Didelphis marsupialis*	Freyer 1999	13.28			stage I
	*Didelphis marsupialis*	Freyer 1999		54		stage II
	*Didelphis marsupialis*	Toeplitz 1920	25		pouch young	stage 0
	*Didelphis marsupialis*	Toeplitz 1920		45.5	pouch young	stage I
	*Didelphis marsupialis*	Toeplitz 1920	10		pouch young	stage II
	*Didelphis marsupialis*	Toeplitz 1920		32.5	pouch young	stage III
	*Didelphis marsupialis*	Toeplitz 1920		19	pouch young	stage IV
	*Caluromys philander*	Denison & Terry 1921		17	embryo	
	*Caluromys philander*	Denison & Terry 1921		17	embryo	
Dasyuromorphia	*Dasyurus viverrinus*	Broom 1909	4	8	pouch young	stage II
	*Dasyurus viverrinus*	Fawcett 1919		7		
	*Dasyurus viverrinus*	Fawcett 1919		9.5		
	*Dasyurus viverrinus*	Fawcett 1919		25		
	*Thylacinus cynocephalus*	Freyer 1999	21.47		juvenile	
Peramelmorphia	*Perameles sp.*	Cords 1915		42	pouch young	
	*Perameles obesula (Isoodon obesulus)*	Esdaile 1916	6.5	12.25		stage I
	*Perameles obesula (Isoodon obesulus)*	Esdaile 1916	6	15.5		stage II
	*Perameles obesula (Isoodon obesulus)*	Esdaile 1916	7	16		stage III
	*Perameles nasuta*	Esdaile 1916	11	23		stage IV
	*Perameles nasuta*	Esdaile 1916	18.5	35		stage V
	*Perameles nasuta*	Esdaile 1916	26	45		stage VI
Diprotodontia	*Trichosurus vulpecula*	Broom 1909		8.5–11	embryo	stage I, A
	*Trichosurus vulpecula*	Broom 1909		10	embryo	stage I, B
	*Trichosurus vulpecula*	Broom 1909		8.5–11	embryo	stage I, C
	*Trichosurus vulpecula*	Broom 1909		8.5–11	embryo	stage I, E
	*Trichosurus vulpecula*	Broom 1909		14	pouch young	stage II
	*Wallabia rufogrisea*	Müller 1986	12	37	pouch young	
	*Vombatus ursinus*	Klutzny 1994	14	31.5	embryo	
Cingulata	*Dasypus novemcinctus*	Reinbach 1952		40	embryo	A
	*Dasypus novemcinctus*	Reinbach 1952		70	embryo	B
	*Tatusia novemcincta (Dasypus novemcinctus)*	Fawcett 1919		60	embryo	
	*Tatusia novemcincta (Dasypus novemcinctus)*	Fawcett 1918		17	embryo	
Afrosoricia	*Hemicentes semispinosus*	Schunke & Zeller 2010	14		embryo	
	*Hemicentetes semispinosus*	Schunke & Zeller 2010	23		embryo	
	*Hemicentetes semispinosus*	Schunke & Zeller 2010	23		embryo	
	*Potamogale velox*	Schunke & Zeller 2010	20		embryo	
	*Potamogale velox*	Schunke & Zeller 2010	25		embryo	
	*Setifer setosus*	Roux 1947		9	embryo	
	*Setifer setosus*	Roux 1947		9.3	embryo	
	*Setifer setosus*	Roux 1947		9.8	embryo	
	*Setifer setosus*	Roux 1947		10.5	embryo	
	*Setifer setosus*	Roux 1947		20.2	embryo	
	*Setifer setosus*	Roux 1947		20.4	embryo	
	*Setifer setosus*	Roux 1947		47.4	embryo	
	*Eremitalpa granti*	Roux 1947		18	embryo	
	*Eremitalpa granti*	Roux 1947		21	embryo	
	*Eremitalpa granti*	Roux 1947		24	embryo	
	*Eremitalpa granti*	Roux 1947		25	embryo	
	*Eremitalpa granti*	Roux 1947		ca. 27	embryo	
	*Eremitalpa granti*	Roux 1947		28.5	embryo	
	*Eremitalpa granti*	Roux 1947		28.5	embryo	
	*Eremitalpa granti*	Roux 1947		31	embryo	
	*Eremitalpa granti*	Roux 1947		36	embryo	
	*Eremitalpa granti*	Roux 1947		41	embryo	
	*Eremitalpa granti*	Roux 1947		45	embryo	
Macroscelidea	*Macroscelides proboscelides*	Ihlau 2011		18.5	embryo	
	*Macroscelides proboscelides*	Ihlau 2011		30.5	embryo	
	*Macroscelides proboscelides*	Ihlau 2011		19.5		dpn 2
	*Macroscelides proboscelides*	Ihlau 2011		40.8	adult	
Tubulidentata	*Orycteropus afer*	Stößel et al. 2010	58	105	fetus	
Hyracoidea	*Procavia capensis*	Stößel et al. 2010	20	42	fetus	
	*Procavia capensis*	Stößel et al. 2010	43	80	fetus	
Proboscidea	*Loxodonta africana*	Stößel et al. 2010	15	32	fetus	
	*Loxodonta africana*	Stößel et al. 2010	58	147	fetus	
Sirenia	*Halicore dugong*	Matthes 1921		15	embryo	
Lagomorpha	*Lepus cuniculus (Oryctolagus cuniculus)*	Voit 1909		45	embryo	
	*Oryctolagus cuniculus*	Mess 1999a	46	150	juvenile	
	*Lepus capensis*	Eloff 1950		46	embryo	
Rodentia	*Castor fiber*	Frahnert 1998	41	114	embryo	
	*Castor fiber*	Frahnert 1998	ca. 63	156	embryo	
	*Sciurus vulgaris*	Frahnert 1998	ca. 10	23	embryo	
	*Sciurus vulgaris*	Frahnert 1998	13	30	embryo	
	*Sciurus vulgaris*	Frahnert 1998	ca. 13	33	embryo	
	*Sciurus vulgaris*	Frahnert 1998	19	44	embryo	
	*Sciurus vulgaris*	Frahnert 1998	38	81	juvenile	
	*Aplodontia rufa*	Frahnert 1998	26	ca. 60	neonate	
	*Phodopus sungorus*	Hauck 1987	11.5	25		dpn 1
	*Octodon degus*	Mess 1997	11	18	fetus	stage I
	*Octodon degus*	Mess 1997	14	22	fetus	stage II
	*Octodon degus*	Mess 1997	24	50	fetus	stage III
	*Octodon degus*	Mess 1997	31	61	neonate	stage IV
	*Octodontomys gliroides*	Mess 1997	19	28	fetus	
	*Aconaemys fuscus*	Mess 1997	22	30	fetus	
	*Spalacopus cyanus*	Mess 1997	13	20	fetus	stage I
	*Spalacopus cyanus*	Mess 1997	14	22.5	fetus	stage II
	*Spalacopus cyanus*	Mess 1997	19	34	fetus	stage III
	*Phodopus sungorus*	Hauck 1987	11.5	25		dpn 1
	*Microtus amphibius (Arvicola amphibius)*	Fawcett 1917		25		
	*Microtus amphibius (Arvicola amphibius)*	Fawcett 1917		17.5		
	*Sciurus vulgaris*	Mess 1999b		30		
	*Sciurus vulgaris*	Frahnert 1998	ca. 10	23	embryo	
	*Sciurus vulgaris*	Frahnert 1998	13	30	embryo	
	*Sciurus vulgaris*	Frahnert 1998	ca. 13	33	embryo	
	*Sciurus vulgaris*	Frahnert 1998	19	44	embryo	
	*Sciurus vulgaris*	Frahnert 1998	38	81	embryo	
	*Petromus typicus*	Mess 1999b	34	92	juvenile	
	*Tupaia belangeri*	Mess 1999b		12.4	embryo	dpc 24
	*Erethizon dorsatus*	Struthers 1927		26	embryo	
	*Peromyscus maniculatus*	Ruf 2004	7.5	12.5	fetus	stage I
	*Peromyscus maniculatus*	Ruf 2004	11.5	21	fetus	stage II
	*Peromyscus maniculatus*	Ruf 2004	14.5	25	neonate	stage III
	*Peromyscus maniculatus*	Ruf 2004	25	65	juvenile	stage IV
	*Peromyscus maniculatus*	Ruf 2004	11	16	fetus	
	*Acomys sp.*	Ruf 2004	20	41	fetus	spec. 1
	*Acomys sp.*	Ruf 2004	22	45	neonate	spec. 2
	*Jaculus jaculus*	Ruf 2004	14	29	fetus	
	*Galea musteloides*	da Silva Neto 2000	14		fetus	stage I
	*Galea musteloides*	da Silva Neto 2000	22		fetus	stage II
	*Kerodon rupestris*	da Silva Neto 2000	28.9		fetus	
Scandentia	*Ptilocercus lowii*	Ruf et al. 2015	17.7	30	embryo	
	*Tupaia glis*	Maier 1980			neonate	
Chiroptera	*Miniopterus schreibersi*	Fawcett 1919		17	embryo	
Primates	*Galago senegalensis*	Warich 1986			fetus	
	*Galago demidovii*	Maier 1980			fetus	
	*Microcebus murinus*	Sorg 1986	ca. 18		fetus	
	*Daubentonia madagascariensis*	Maier & Ruf 2014	41	98	fetus	
	*Papio hamadryas*	Reinhard 1958	12.5	33	embryo	
	*Pan troglodytes*	Starck & Kummer 1962		71	embryo	
	*Homo*	Bersch & Reinbach 1970		52	embryo	
	*Homo*	Grube & Reinbach 1976		80	embryo	
	*Homo*	Maier & Ruf 2014	63		fetus	
Eulipotyphla	*Erinaceus europaeus*	Fawcett 1918		25		
	*Erinaceus europaeus*	Fawcett 1918		19	embryo	
	*Erinaceus europaeus*	Michelsson 1922				
	*Erinaceus europaeus*	Michelsson 1922				
	*Erinaceus europaeus*	Michelsson 1922				
	*Erinaceus europaeus*	Michelsson 1922				
	*Erinaceus europaeus*	Michelsson 1922				
	*Talpa europaea*	Fawcett 1918		19	embryo	
	*Talpa europaea*	Jacobson 1928			24 embryos	
	*Talpa europaea*	Fischer 1901			several embryos	
Soricomorpha	*Suncus orangiae*	Roux 1947		6	embryo	
	*Suncus orangiae*	Roux 1947		6.4	embryo	
	*Suncus orangiae*	Roux 1947		6.8	embryo	
	*Suncus orangiae*	Roux 1947		7.2	embryo	
	*Suncus orangiae*	Roux 1947		8.4	embryo	
	*Suncus orangiae*	Roux 1947		9.5	embryo	
	*Suncus orangiae*	Roux 1947		15.6	embryo	
	*Suncus orangiae*	Roux 1947		18.3	embryo	
	*Suncus orangiae*	Roux 1947		22	embryo	
	*Suncus orangiae*	Roux 1947		23.5	embryo	
	*Suncus orangiae*	Roux 1947		28	embryo	
	*Neomys fodiens*	Maier 2002			young adult	
	*Sorex araneus*	Maier 2002			young adult	
Artiodactyla	*Alces aleces*	Pinus 1928		22	embryo	
	*Bos taurus*	Fawcett 1918		40	embryo	
	*Bos taurus*	Fawcett 1918		19	embryo	
Perissodactyla	*Sus scorfa*	Parker 1874			embryo	stage I
	*Sus scorfa*	Parker 1874			embryo	stage II
	*Sus scorfa*	Parker 1874			embryo	stage III
	*Sus scorfa*	Parker 1874			embryo	stage IV
	*Sus scorfa*	Parker 1874			embryo	stage V
	*Sus scorfa*	Parker 1874			embryo	stage VI
	*Sus scorfa*	Parker 1874			neonate	stage VII
	*Sus scorfa*	Parker 1874			juvenile	
	*Sus scorfa*	Parker 1874			adult	stage IX
	*Equus caballus*	von Mering 1994		32		
	*Equus caballus*	von Mering 1994		36		stage II
	*Equus caballus*	von Mering 1994		40		stage III
	*Equus caballus*	von Mering 1994		44		stage IV
	*Equus caballus*	von Mering 1994		77		stage V
	*Equus caballus*	von Mering 1994		87		stage VI
	*Equus caballus*	von Mering 1994		120		stage VII
	*Equus caballus*	von Mering 1994		150		stage VIII
	*Equus sp.*	von Mering 1994	60	170		stage IX
Carnivora	*Poecilophoca weddelli (Leptonychotes weddellii)*	Fawcett 1918		27	embryo	
	*Cryptoprocta ferox*	Köhncke 1985		54	fetus	ca. 6 weeks
	*Cryptoprocta ferox*	Köhncke 1985		57	fetus	ca. 6 weeks
	*Felis catus*	Terry 1917		23.1	embryo	
	*Felis catus*	Terry 1917		10	embryo	
	*Felis catus*	Terry 1917		12	embryo	
	*Felis catus*	Terry 1917		15	embryo	
	*Felis catus*	Terry 1917		17	embryo	
	*Felis catus*	Terry 1917		20	embryo	
	*Felis catus*	Terry 1917		24	embryo	
	*Felis catus*	Terry 1917		30	embryo	
	*Felis catus*	Terry 1917		35	embryo	
	*Canis lupus familiars*	Olmstead 1911		27	embryo

## Appendix 1

Documentation on the variability of ontogenetic characters in marsupial species studied herein.

*Caluromys philander* ESUT-C6

1_1_1_1_0_•_1_0_1_0_1_•_0_2_1_0_1_•_1_2_1_1_0_•_0_0_0_0_0_•_0_1_1?_0_1_•_1_1_1_1_0_•_0_0_?_1?_1?_•_?_0_0

*Caluromys philander* ESUT-C15

1_1_1_1_0_•_1_0_1_1_1_•_0_2_1_0_1_•_1_2_1_1_0_•_0_0_0_0_0_•_0_1_?_0_1_•_0_1_1_1_0_•_0_1_0_1_1?_•_?_0_0

*Caluromys philander* ESUT-C13

1_0_1_1_1_•_0_2_1_1_1_•_1_1_1_0_1_•_1_0_0_1_0_•_0_1_2_0_1_•_0_1_0_0_1_•_1_1_1_1_0_•_0_1_1_1_1_•_1_1_0

*Caluromys philander* ESUT-C25

1_0_1_1_1_•_0_2_1_1_1_•_1_?_1_1_1_•_1_0_?_1_0_•_?_2_?_?_?_•_?_1_?_0_1_•_?_1_1_1_0_•_0_1_0_1_1_•_1_1?_0

*Caluromys philander* ESUT-C26

1_0_1_1_1_•_0_2_1_1_1_•_1_1_1_1_1_•_1_0_0_1_0_•_0_2?_2_0_1_•_0_1_1?_0?_1_•_1_1_1_1_0_•_0_1_1_1_1_•_1_1_0

*Macropus eugenii* ESUT-M11

1_?_1_1_1_•_2_1_1_0_1_•_1_2_1_0_1_•_1_2_1_1_1_•_1_2?_1_0_0_•_0_1_1_1_0_•_1_1_1_1_0_•_0_1_1_1_1_•_?_0_0

*Macropus eugenii* ESUT-M16

1_?_1_1_1_•_2_2_1_0_1_•_1_2_1_0_1_•_1_2_0_1_1_•_1_2?_1_0_1_•_0_1_0_1_0_•_1_1?_1?_1_0_•_0_1_1_1_1_•_?_?_0

*Macropus eugenii* ESUT-M28

1_0_1_1_1_•_1_2_1_1_1_•_1_2_1_0_1_•_1_2_1_1_1_•_1_2?_1_0_1_•_0_1_0_1_2_•_1_1_1_1_0_•_0_1_1_1_1_•_?_?_0

*Macropus eugenii* ESUT-M53

1_0_1_1_0_•_1_0_1_1_1_•_1_2_1_0_1_•_2_2_1_1_1_•_1_2?_1_1_1_•_0_1_0_1_2_•_1_0_1_0_0_•_0_1_1_1_1_•_?_?_0

*Monodelphis domestica* ESUT-Mo10.5

1_1_1_1_0_•_0_2_1_1_1_•_0_0_1_0_0_•_1_2_1_1_1_•_1_2_2_0_0_•_0_1_1_1_1_•_0_0?_0?_1_0_•_0_1_0_?_0_•_?_0_0

*Monodelphis domestica* ESUT-Mo11.5

1_1_1_1_2?_•_0_2_1_1_0?_•_?_1_1_1_0_•_1_2_1_1_0_•_0_0_0_0_0_•_0_1_1_1_1_•_0_1_1_1_0_•_0_1_1_1_0_•_?_0_0

*Monodelphis domestica* ESUT-Mo8.5

1_0_1_1_1?_•_0_2_1_1_1_•_0_0_1_1_0_•_1_2_0_1_0_•_0_2_2_0_0_•_0_1_1_1_1_•_0?_1_1_1_0_•_0_1_1_1_1_•_1_1_0

*Monodelphis domestica* ESUT-Mo63

1_0_1_1_1_•_0_2_1_1_1_•_0_0_1_1_0_•_1_2_0_1_0_•_0_2_2_0_1_•_0_1_1_1_1_•_1_1_1_1_0_•_0_1_1_1_1_•_1_1_0

*Sminthopsis virginiae* AMNH SR 1A

1_?_1_1_2?_•_1_1_1?_1_1_•_1_1_1_0_0_•_1_2_0_1_1_•_0_1_1_0_?_•_?_1_1_1_1?_•_0?_1_1_1_0_•_0_0_?_0_0_•_?_0_0

*Sminthopsis virginiae* AMNH SR 2A

1_?_1_1_1?_•_1_1_1_1_1_•_1_1_1_0_0_•_1_2_1_1_1_•_1_2_1_0_0_•_0_1_?_1_0?_•_0?_1_1_1_0_•_0_1_0_0_0_•_1?_0_0

*Sminthopsis virginiae* AMNH SR 3A

1_0_1_1_1_•_0_2_1_1_1_•_1_1_1_0_0_•_1_2_1_1_2_•_1_2_2_0_0_•_1_1_?_1_1_•_1_1_1_1_0_•_0_1_1_1_1_•_1_0?_0

*Sminthopsis virginiae* AMNH SR 4A

1_0_1_1_1_•_0_2_1_1_1_•_1_1_1_0_0_•_1_2_0_1_2_•_0_2_2_1_1_•_1_1_?_1_?_•_?_1_1_1_0_•_0_1_1_1_1_•_1_1_0

## Appendix 2

Data matrix on characters of the cupula nasi anterior and the anterior nasal capsule and references for the species information. ? = unknown state; 0?, 1?, 2? = character tending to this state; polymorphic character in “[ ]”. Note that all marsupial embryos studied herein are separately coded in this list, but they were included to the analysis as one terminal taxon per species with polymorphism reflecting ontogenetic variability.

*Acomys dimidiatus* LANE-Aco 21 [this study]

1?_?_1_?_?_•_?_?_1_0_1_•_?_?_1_?_?_•_?_?_?_1_1_•_1_1_1_?_1_•_0_1_?_?_?_•_0_?_?_?_1_•_1_1_1_?_?_•_?_?_0

*Acomys sp.* [28]

1_0_?_?_?_•_0_?_1_?_0_•_1_?_1_?_?_•_?_?_?_1_?_•_?_?_?_?_?_•_?_?_?_?_?_•_0_1_?_?_?_•_1_0_?_1_1_•_1_?_?

*Aconaemys fuscus* [22]

1_?_?_1_1_•_?_?_?_?_0_•_?_?_?_?_?_•_?_?_?_0_?_•_?_?_?_?_?_•_?_1_?_?_0_•_?_?_?_?_1_•_?_?_?_?_?_•_?_?_?

*Alces alces* [83]

1_?_?_?_?_•_?_?_1_?_?_•_?_?_?_?_?_•_?_?_?_1_0_•_?_0_1_?_?_•_1_1_?_?_?_•_?_?_1_?_0_•_?_1_0_?_1_•_1_?_?

*Aplodontia rufa* [38]

1_1_?_1_2_•_0?_[0_2]_1_1_1_•_1_1?_1_?_?_•_1_2_?_1_?_•_0_1_?_?_?_•_?_?_0_1_2_•_?_1_1_1_1_•_0_1_[0_1]_1_?_•_1?_?_0

*Arvicola (*“*Microtus*”) *amphibius* [57]

1_?_?_0_?_•_?_?_?_1_1_•_?_0_1_?_?_•_?_?_?_1_?_•_?_1_1_?_?_•_?_1_?_?_?_•_?_1_1_1_1_•_1_1_?_?_1_•_1_?_?

*Atelerix_albiventris* LANE-Atx25 [this study]

1_?_1_1_0_•_?_?_1_1_1_•_1_1_1_1_0_•_1_1_?_?_?_•_?_?_?_?_?_•_?_1_1_?_?_•_0_1_1_1_1_•_0_1_?_1_?_•_?_?_0

*Bos taurus* [6]

1_?_?_0_?_•_?_?_1_?_?_•_?_?_?_?_?_•_?_0_?_1_1_•_?_1_2_?_?_•_?_?_?_?_?_•_?_?_?_?_?_•_1_?_?_?_?_•_?_?_?

*Caluromys philander* [47]

1_0_1_1_?_•_?_?_?_?_?_•_?_?_?_1_1_•_1_0_2_?_?_•_?_?_?_?_?_•_?_1_?_?_?_•_1_?_?_?_?_•_?_1_1_?_?_•_1_?_0

*Caluromys philander* [this study], for individual specimen coding see Appendix 1

1_[0_1]_1_1_[0_1]_•_[0_1]_[0_2]_1_[0_1]_1_•_[0_1]_[1_2]_1_[0_1]_1_•_1_[0_2]_[0_1_2]_1_0_•_0_[0_1_2]_[0_2]_0_[0_1]_•_0_1_[0_1]_0_1_•_[0_1]_1_1_1_0_•_0_[0_1]_[0_1]_1_1_•_1_[0_1]_0

*Canis lupus familiaris* [84]

?_?_?_0_?_•_?_?_1_1_?_•_?_?_?_?_?_•_0_0_?_1_1_•_?_?_?_?_?_•_?_1_0_?_?_•_?_1_1_1_?_•_0_1_1_?_1_•_1_?_?

*Castor fiber* [38]

1_1_1_1_2_•_2_?_1_1_1_•_1_1_1_1_1_•_1_2_1_1_?_•_1_1_1_?_?_•_?_1_1?_2?_1_•_?_0_0_1_0_•_0_1_0_1_1_•_1_?_0

*Cryptoprocta ferox* [15]

1_?_?_0_?_•_?_?_1_0_1_•_?_0_1_1_?_•_2_[0_1]_?_1_0_•_1_0_0_?_?_•_?_?_?_?_1_•_?_1_0?_1_0_•_0_1_?_1_1_•_1_?_?

*Dasypus novemcinctus* “*Tatusia novemcinctus*” [31–33]

1_?_?_0_0_•_?_?_1_1_1_•_1_2_0_?_?_•_0_0_2_1_1_•_?_?_?_?_0_•_0_1_0_?_1_•_0_1_1_1_?_•_1_1_?_1_1_•_1_?_0

*Dasyurus viverrinus* [31, 37]

1_1_?_1_[1_2]_•_1_[0_2]_1_1_1_•_1_1_1_1_1_•_1_2_1_[0_1]_?_•_?_?_?_?_?_•_?_1_1_?_?_•_?_?_1_?_0_•_0_1_?_?_?_•_?_?_0

*Daubentonia madagascariensis* [4]

1_?_?_?_?_•_?_?_1_?_?_•_?_?_?_?_?_•_?_?_?_1_?_•_?_?_?_?_?_•_?_1_?_1_0_•_?_?_?_?_?_•_?_1_?_1_1_•_1_?_?

*Didelphis marsupialis* [36, 42]

1_?_1_1_1_•_1_?_1_1_1_•_0_1_1_?_1_•_1_2_?_1_1_•_?_1_1_?_?_•_0_1_?_1_1_•_?_1_1_1_0_•_?_1_1_0_1_•_1_?_0

*Echinops telfairi* LANE-Ech7a [this study]

1?_?_?_?_?_•_?_?_1?_1?_?_•_?_?_1?_?_1_•_?_?_?_?_?_•_?_?_?_?_?_•_?_1_?_?_?_•_?_1_?_?_?_•_?_?_?_?_?_•_?_?_?

*Equus caballus* [85]

1_1_?_1?_1_•_?_2_?_?_?_•_?_?_?_?_?_•_1_?_?_?_?_•_?_?_?_?_?_•_?_1_?_?_?_•_?_?_?_?_?_•_?_1_?_1_?_•_?_?_?

*Eremitalpa granti* [30]

0?_1_0_0_1_•_0_0_1_1_1_•_1_?_0_?_?_•_0_0_?_1_?_•_?_?_?_?_?_•_?_1_?_1_1_•_0?_1_1_1_0_•_1_1_1_?_1_•_1_?_?

*Erethizon dorsatum* [86]

1_?_?_1_?_•_?_?_?_?_?_•_?_?_?_?_?_•_1_?_0_?_?_•_?_?_?_?_?_•_?_1_?_?_?_•_?_1_1_1_1_•_?_1_1_?_1_•_1_?_?

*Erinaceus europaeus* [45]

1_?_1_1_1_•_?_?_1_1_1_•_1_1_1_?_?_•_1_2_?_1_1_•_1_1_1_?_?_•_?_1_?_?_1_•_1_1_1_1_1_•_1_1_0_?_1_•_1_?_?

*Felis catus* [87]

?_?_?_?_?_•_?_?_?_?_?_•_?_?_?_?_?_•_2_?_0_?_?_•_?_?_?_?_?_•_?_1_?_?_?_•_?_1_1_1_?_•_?_1_1_?_1_•_1_?_?

*Galago demidovii* [24]

1_?_?_1_?_•_?_?_?_?_?_•_?_?_?_?_?_•_1_?_?_1_?_•_?_?_?_?_?_•_?_1_?_?_?_•_1_?_0_?_?_•_?_?_?_1_1_•_1_?_?

*Galago senegalensis* [55]

1?_?_?_1_1_•_?_?_1_?_0_•_1_?_1_1_1_•_1_2_0_1_0?_•_?_1_1_?_?_•_?_1_?_?_1_•_0_0_0_1_?_•_0_1_?_1_1_•_1_?_0

*Galea musteloides* [49]

1_?_?_?_1_•_?_?_1_?_0?_•_1_?_?_?_?_•_?_?_?_0_?_•_?_?_?_?_?_•_?_1_?_?_2_•_?_1_1_1_?_•_?_1_?_1_1_•_1_?_?

*Halicore dugong* [14]

?_?_?_0_?_•_?_?_?_?_?_•_?_?_?_?_?_•_?_?_?_?_?_•_?_?_?_?_?_•_?_1_?_?_?_•_?_?_?_?_?_•_?_1_?_?_?_•_?_?_?

*Hemicentetes semispinosus* [39]

1_0_?_0_1_•_?_?_1_1_1_•_1_1_1_?_0_•_1_0_?_1_?_•_?_1_1_?_1_•_?_?_?_?_2_•_?_1_1_1_0_•_1_1_0_?_?_•_?_?_1

*Homo_sapiens* [4, 43, 44]

1_?_0_0_1_•_?_?_?_0_?_•_?_2_1_?_?_•_0_?_?_1_?_•_?_?_?_?_0_•_0_0_?_?_?_•_?_?_?_?_?_•_?_1_?_?_?_•_1_?_0

*Isoodon_obesulus* “*Perameles obesula*” [41]

1_?_?_?_2_•_?_?_1?_1?_1_•_?_1_1_?_?_•_2_?_1_1_1_•_?_1_1_?_0_•_0_1_1_?_?_•_0_1_1_1_?_•_0_1_?_0_0_•_0_?_0

*Jaculus jaculus* [28]

1_?_0_0_?_•_?_?_1_1_1_•_0_?_1_?_?_•_?_?_?_1_?_•_1_?_1_?_0_•_?_?_?_?_0_•_?_1_1_1_1_•_0_0_?_1_1_•_1_?_?

*Kerodon rupestris* [49]

1_?_?_?_0_•_?_?_1_?_0?_•_1_?_?_?_?_•_?_?_?_1_?_•_?_?_?_?_?_•_?_1_?_?_0_•_?_1_1_1_?_•_?_1_?_1_1_•_1_?_?

*Lepus capensis* [13]

?_?_?_0_?_•_?_?_?_?_?_•_?_?_?_?_?_•_?_?_?_?_?_•_?_?_?_?_?_•_?_1_?_?_?_•_?_0_0_1_?_•_1_?_?_?_1_•_?_?_?

*Loxodonta africana* [25]

0?_1_1_?_0_•_?_?_0_?_?_•_?_?_1_?_?_•_3_?_?_?_?_•_?_?_?_?_?_•_?_?_?_?_?_•_?_?_?_?_0_•_?_?_?_1_?_•_?_?_?

*Macropus eugenii* [this study], for individual specimen coding see Appendix 1

1_0_1_1_[0_1]_•_[1_2]_[0_1_2]_1_[0_1]_1_•_1_2_1_0_1_•_[1_2]_2_[0_1]_1_1_•_1_2_1_[0_1]_[0_1]_•_0_1_[0_1]_1_[0_2]_•_1_[0_1]_1_[0_1]_0_•_0_1_1_1_1_•_?_0_0

*Macroscelides proboscelides* [48]

1_?_?_?_1_•_?_?_1_?_?_•_?_?_1_0_?_•_2_?_?_1_?_•_?_?_?_?_?_•_?_?_?_?_?_•_?_1_1_?_1_•_?_1_?_1_1_•_1_?_?

*Microcebus murinus* [88]

1_0_?_1_1_•_?_?_1_?_0_•_1_?_1_1_?_•_1_2_2_1_0_•_?_1_1_?_?_•_?_1_0_?_0?_•_1_1?_1?_1_?_•_0_1_?_1_1_•_1_?_0

*Miniopterus schreibersi* [31]

1?_0_0_0_1_•_0_1_1_1_0_•_?_2_1_1_1_•_0_?_?_1_0_•_1_0_0_?_?_•_?_1_0_?_0_•_0_0_0_1_1_•_?_1_1_1_1_•_?_?_1

*Monodelphis domestica* [36]

1_?_1_1_1_•_?_0_?_?_1_•_?_0_1_?_?_•_1_2_?_1_?_•_?_2_2_?_?_•_1_1_?_1_?_•_?_1_1___0_•_?_?_?_1_1_•_1_?_0

*Monodelphis domestica* [this study], for individual specimen coding see Appendix 1

1_[0_1]_1_1_[0_1_2]_•_0_[0_2]_1_1_[0_1]_•_0_[0_1]_1_[0_1]_0_•_1_2_[0_1]_1_[0_1]_•_[0_1]_[0_2]_[0_2]_0_[0_1]_•_1_1_1_1_1_•_[0_1]_[0_1]_[0_1]_1_0_•_0_1_[0_1]_1_[0_1]_•_1_[0_1]_0

*Neomys fodiens* [56]

1_?_?_?_1_•_?_?_?_?_?_•_?_?_1_?_?_•_1_?_?_1_?_•_?_0_0_?_?_•_?_1_?_?_?_•_?_?_1_0_1_•_1_1_?_1_1_•_?_?_?

*Octodon degus* [22]

1_0_0_0_1_•_0_0_1_0_0_•_1_2?_1_0_?_•_0_0_?_1_1_•_0_1_1_?_?_•_0_1_?_2_0_•_0_?_1_1_1_•_?_1_?_1_1_•_1_?_0

*Octodontomys gliroides* [22]

1_0_0_0_1_•_?_?_1_1_0_•_?_0_1_0_0_•_0_?_?_1_?_•_?_?_?_?_0_•_?_1_?_?_0_•_?_?_1_1_1_•_?_?_?_1_1_•_1_?_0

*Orycteropus afer* [25]

1?_?_0_0_0_•_?_?_1_1_0_•_?_1_1_?_?_•_?_?_?_?_?_•_?_?_?_?_?_•_?_?_?_?_?_•_?_?_0_?_1_•_?_?_?_1_?_•_1_?_?

*Oryctolagus* (“*Lepus*”) *cuniculus* [8, 12]

1_?_0_0_0_•_?_1_0_?_?_•_?_2_?_?_?_•_0_?_?_1_?_•_?_?_?_?_?_•_?_1_?_?_?_•_?_0_0_1_?_•_1_1_?_1_1?_•_?_?_?

*Pan_troglodytes* [7]

?_?_?_?_?_•_?_?_?_?_0_•_?_?_?_?_?_•_?_?_?_?_?_•_?_?_?_?_?_•_?_0_?_?_?_•_?_0_?_?_?_•_?_?_?_?_?_•_1_?_?

*Papio hamadryas* [16]

0_?_?_0_?_•_?_?_1_?_?_•_1_2_?_?_?_•_0_?_?_1_?_•_?_?_?_?_?_•_?_1_?_?_?_•_?_0_0_1_?_•_?_1_1_?_?_•_1_?_?

*Perameles nasuta* [41]

1_?_?_?_2_•_?_?_1_1_0_•_?_1_?_?_?_•_2_?_1_1_1_•_?_?_?_?_0_•_0_1_1_?_?_•_?_?_1_?_0_•_0_1_?_?_?_•_1_?_0

*Perameles sp.* [40]

1_?_?_0_1_•_?_?_?_?_?_•_?_1_0_?_?_•_2_0_1_1_?_•_?_?_?_0_?_•_?_1_1_?_1_•_1_1_1_1?_0_•_0_1_0?_?_1_•_1_?_?

*Peromyscus maniculatus* [28]

1_0_1_1_1_•_0_?_1_1_?_•_1_0?_1_1_1_•_1_0_?_1_?_•_1_2_1_?_1_•_1_1_?_1_2_•_1_1_1_1_?_•_1_1_0_1_1_•_1_?_?

*Petaurus breviceps* LANE-P82A [this study]

1_1_1_1_1_•_1?_0_1_0_?_•_0?_2_1_0_1_•_1_2_1_1_1_•_1_2_1_0_1_•_0_1_?_2_?_•_0_1?_1_1_0_•_0_1_?_0_0_•_0_?_0

*Petromus typicus* [18]

1_?_?_1_1_•_?_?_1_?_0_•_?_?_?_?_?_•_0_?_?_1_?_•_?_?_?_?_?_•_?_?_?_2_?_•_?_?_1_?_0_•_?_?_?_?_1_•_?_?_?

*Phodopus sungorus* [46]

1_?_0_0_?_•_0_?_1_?_1_•_1_?_1_0_?_•_0_?_?_1_0_•_?_?_?_?_?_•_1_1_?_?_?_•_?_1_1_1_0_•_?_1_?_1_1_•_1_?_?

*Leptonychotes* (“*Poecilophoca*”) *weddellii* [6]

?_?_?_?_?_•_?_?_?_?_?_•_?_0_?_?_?_•_?_?_?_1_?_•_?_?_?_?_?_•_?_?_?_?_?_•_?_?_0_1_0_•_1_1_?_?_?_•_1_?_?

*Potamogale velox* [39]

1_1_?_1_?_•_?_?_?_?_?_•_1_?_1_1_?_•_1_?_?_?_?_•_?_?_?_?_?_•_?_1?_?_?_?_•_?_0_?_?_0_•_1_1_1_?_?_•_?_?_?

*Procavia capensis* [25]

1_?_?_1_1_•_?_?_?_?_?_•_?_?_?_?_?_•_1_?_?_1_?_•_?_?_?_?_?_•_?_?_?_?_?_•_?_?_?_?_0_•_?_?_?_?_1_•_1_?_1

*Ptilocercus lowii* [89]

1_?_?_?_?_•_?_?_?_?_?_•_?_?_1_?_?_•_?_?_?_1_?_•_?_?_?_?_?_•_?_1_?_?_?_•_?_?_1_?_?_•_1_1_?_1_1_•_1_?_?

*Sciurus vulgaris* [38]

1_1_?_?_1_1_•_?_?_1_?_1_•_?_?_1_1_?_•_1_?_?_1_?_•_?_?_?_?_?_•_?_?_?_1_?_•_?_?_1_1_?_•_?_?_?_?_?_•_?_?_?

*Sciurus vulgaris* [18]

2_1_1_?_1_2_•_1_0_1_0_1_•_1_2_1_0?_0_•_1_0_1_1_1_•_0_1_?_1?_?_•_?_1_?_1_0_•_?_1_1_1_1_•_1_?_?_1_1_•_1_?_0

*Setifer setosus* [30]

1_1_1_1_1_•_1_0_1_1_1_•_1_1_1_1_1_•_1_?_?_1_?_•_?_?_1_?_?_•_?_?_?_0_0_•_?_1_1_1_0_•_1_1_1_?_1_•_1_?_?

*Sminthopsis virginiae* [this study], for individual specimen coding see Appendix 1

1_0_1_1_[1_2]_•_[0_1]_[1_2]_1_1_1_•_1_1_1_0_0_•_1_2_[0_1]_1_[1_2]_•_[0_1]_[1_2]_[1_2]_[0_1]_[0_1]_•_[0_1]_1_1_1_[0_1]__•_[0_1]_1_1_1_0_•_0_[0_1]_[0_1]_[0_1]_[0_1]_•_1_[0_1]_0

*Sorex araneus* [56]

1_?_?_?_1_•_?_?_?_?_?_•_?_?_1_?_?_•_1_?_?_1_?_•_?_0_0_?_?_•_?_?_?_?_?_•_?_1_?_?_?_•_1_1_?_?_?_•_?_?_?

*Spalacopus cyanus* [22]

1_?_?_0_1_•_?_?_?_?_0_•_?_2?_1_0_?_•_0_0_?_1_?_•_0_?_?_?_?_•_?_1_?_2_?_•_?_?_?_?_1_•_?_?_?_?_?_•_?_?_?

*Suncus orangiae* [30]

1_?_?_1_1_•_0_0_1_1_1_•_1_1_1_1_1_•_1_?_?_1_?_•_1_?_1_?_?_•_?_1_1_?_?_•_?_1_1_1_0_•_1_1_0?1_?_1_•_1_?_?

*Sus scorfa* [53]

1_?_?_1_1_•_?_2_1_1_1_•_1_1_1_?_?_•_1_?_?_1_?_•_?_0_?_?_?_•_?_?_?_0_2_•_?_?_?_?_0_•_0_1_?_1_1_•_?_?_1

*Tachyglossus aculeatus* [20]

1_?_?_1_1_•_0_?_?_?_?_•_?_1_1_?_1_•_4_1_?_1_?_•_?_0_1_?_1_•_1_1_?_?_2_•_?_?_1_?_1_•_?_?_?_1_0_•_1_?_0

*Talpa europaea* [6, 10, 50]

1_?_?_?_1_•_?_?_1_?_?_•_?_?_1_1_?_•_?_?_?_1_2_•_?_[1_0]_[1_2]_0_1_•_1?_?_?_?_?_•_?_1_1_1_?_•_1_1_?_?_?_•_1?_?_?

*Thylacinus cynocephalus* [36]

1_1_1_1_1_•_0_?_1_?_1_•_1_?_1_?_?_•_1_?_?_1_1_•_?_1_1_?_?_•_0_1_?_1_?_•_?_1_?_?_?_•_?_1_1_1_1_•_1_?_0

*Trichosurus vulpecula* [36]

1_?_?_1_1_•_?_?_1_0_1_•_?_2_1_1_1_•_1_1_2_1_1_•_1_1_1_0_?_•_?_1_1_?_?_•_0_0?_?_?_0_•_?_1_?_?_?_•_?_?_0

*Tupaia belangeri* [18]

?_?_?_?_1_•_?_?_1_?_?_•_?_?_?_?_?_•_?_?_?_?_?_•_?_?_?_?_?_•_?_?_?_?_?_•_?_?_1_?_?_•_?_?_?_?_?_•_?_?_?

*Tupaia glis* [24]

1_?_?_1_1_•_?_?_?_?_?_•_?_?_1_?_?_•_1_?_?_1_1_•_?_?_?_?_?_•_?_1_?_?_?_•_0_1_1_1_?_•_1_?_?_1_1_•_?_?_?

*Vombatus ursinus* [52]

1_0_?_1_1_•_?_?_1_?_?_•_1_2_1_1_1_•_1_2_2_1_1_•_1_1_1_?_0_•_0_1_0_?_0_•_1_0_1_0_0_•_0_1_0_?_1_•_1_?_0

*Wallabia rufogrisea* [54]

1_0_?_1_1_•_?_?_1_?_1_•_?_2_1_0_?_•_1_2_?_1_1_•_1_1_1_?_?_•_0_1_1_1_0_•_?_1_1_1_0_•_0_1_?_1_1_•_1_?_0

## Abbreviations

ai: Area internarica
ane: Apertura nasi externa
at: Atrioturbinale
cc: Cartilago cupularis
cdn: Cartilago ductus nasopalatine
cna: Cupula nasi anterior
cpa: Cartilago paraseptalis anterior
CRL: Crown-rump length
dnl: Ductus nasolacrimalis
dnp: Ductus nasopaltinus
dpc: Days post conception
dpn: Postnatal days
fia: Fenestra internasalis anterior
fpt: Foramen praetransversale
HL: Head length
lat. lam: Lateral lamina
lta: Lamina transversalis anterior
mat: Marginoturbinale
med. lam: Medial lamina
mt: Maxilloturbinale
pas: Processus alaris superior
pc: Processus cupularis
plv: Processus lateralis ventralis
pmx: Praemaxillare
pn: Paries nasi
ppl: Processus paralacrimalis
sn: Septum nasi
ss: Sulcus supraseptalis
sv: Sulcus ventralis
tea: Tectum nasi anterius
tei: Tectum nasi intermedium
tep: Tectum nasi profundum
tn: Tectum nasi
vno: Organon vomeronasale
vo: Vomer
za: Zona annularis
